# Trehalose in pine wood nematode participates in DJ3 formation and confers resistance to low-temperature stress

**DOI:** 10.1186/s12864-021-07839-0

**Published:** 2021-07-09

**Authors:** Qiaoli Chen, Ruizhi Zhang, Danlei Li, Feng Wang, Shengwei Jiang, Jianan Wang

**Affiliations:** 1 Key Laboratory of Alien Forest Pests Monitoring and Control-Heilongjiang Province, School of Forestry, Northeast Forestry University, 150040 Harbin, Heilongjiang P. R. China; 2 Key Laboratory of Sustainable Forest Ecosystem Management-Ministry of Education, Northeast Forestry University, 150040 Harbin, Heilongjiang P. R. China; 3 Station of Forest and Grassland Pest Control and Quarantine, 110001 Shenyang, Liaoning P. R. China

**Keywords:** Pine wood nematode, Pine wilt disease, Third-stage dispersal juvenile, Trehalose, Low-temperature resistance

## Abstract

**Background:**

Recently, pine wood nematode (PWN, *Bursaphelenchus xylophilus*) has been found in the extreme cold area of northeast China. The third-stage dispersal juvenile (DJ3) of PWN, which is a long-lived stress-resistant stage, plays an important role in the process of PWN spreading to low-temperature areas, as this stage can survive under unfavorable conditions.

**Results:**

Weighted correlation network analysis (WGCNA) was used to analyze the expression patterns of 15,889 genes included in 21 RNA-Seq results of PWN at DJ3 and the other 6 different stages, and a total of 12 coexpression modules were obtained. Among them, the magenta module has the highest correlation with DJ3, which included a total of 652 genes. KEGG enrichment analysis showed that most of the genes in the magenta module were involved in metabolic processes, which were related to autophagy and longevity regulation. These pathways included starch and sucrose metabolism, which contains trehalose metabolism. To explore the function of trehalose in DJ3 formation and survival under − 20 °C, a trehalose-6-phosphate synthase encoding gene (*Bx-tps*), a trehalose-6-phosphate phosphatase encoding gene (*Bx-tpp*) and 7 trehalase encoding genes (*Bx-tre*s) were identified and investigated. The expression of these 9 genes was related to the formation of DJ3. A treatment under − 20 °C induced the accumulation of trehalose. The survival rate of DJ3 at -20 °C reduced after silencing of any of these trehalose metabolism genes. Further analysis suggested that two trehalose synthesis genes were highly correlated with DJ3 and might be involved in autophagy by regulating with energy conversion related genes.

**Conclusions:**

The above results indicated that trehalose metabolism promotes DJ3 formation and helps DJ3 survive at -20 °C. Although trehalose accumulation is favorable for DJ3 to cope with low-temperature stress, multiple trehalose metabolism genes need to work together. There may be a multi-path regulated physiological process involving trehalose synthesis genes under low-temperature stress resistance. This physiological process may regulate the formation and maintenance of DJ3 through autophagy and energy conversion.

**Supplementary Information:**

The online version contains supplementary material available at 10.1186/s12864-021-07839-0.

## Background

The pine wood nematode (PWN, *Bursaphelenchus xylophilus*), a migratory endoparasite, causes pine wilt disease (PWD), which is one of the most damaging issues affecting conifer forests (in particular *Pinus* spp.) [1]. In the past, it was thought that PWN was difficult to survive in the cryogenic environment. Therefore, only little attention has been paid to the study of the nematode low temperature resistance. However, according to an announcement of the State Forestry and Grassland Administration of China (No. 4, 2019), PWN had been found in the northeast areas in China, where the lowest temperature in winter is under − 20 °C, which indicated that the large areas of pine forest in northern China may be at risk of infection, making it more urgent to study the resistance mechanism of this nematode.

**Fig. 1:**
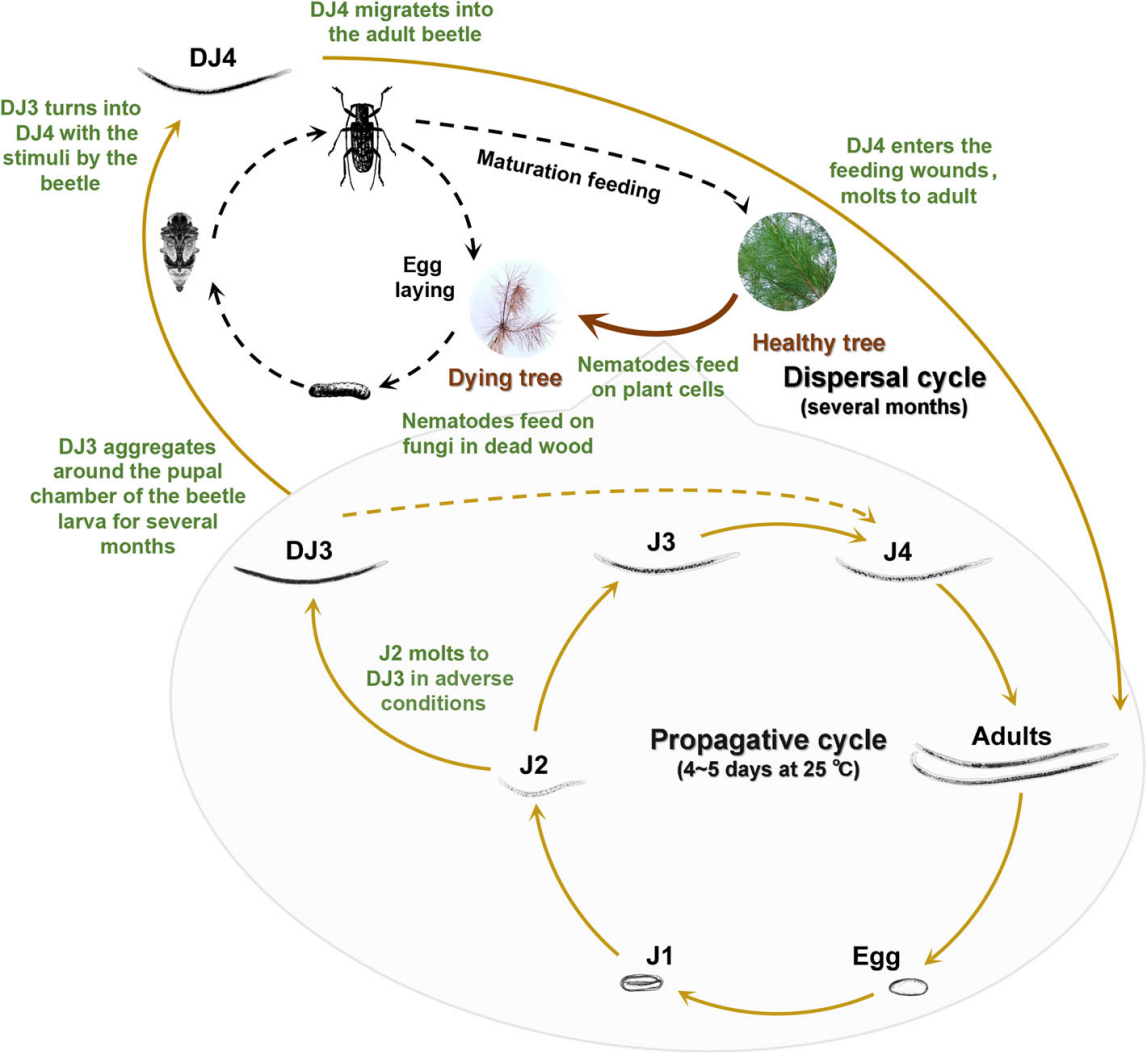
Life cycle of pine wood nematode. J1, first-stage propagative juvenile; J2, second-stage propagative juvenile; J3, third-stage propagative juvenile; J4, fourth-stage propagative juvenile; DJ3, third-stage dispersal juvenile; DJ4, fourth-stage dispersal juvenile. The images depicted in this figure are our original

The spread of PWN to low-temperature areas was most likely due to the fact that unlike many other Aphelenchoididae nematodes, PWN has a complex life cycle, including specific stages of development at which it survives in hostile environments. PWN can have both phytophagous and mycophagous phases of development [2, 3] with distinct life cycles including four propagative and two dispersal juvenile stages (Fig. 1) [4]. During the propagative stage, the population is expanded by egg, juvenile (J1 ~ J4) and adult. At the end of summer and the beginning of autumn, drought, cold, food shortage and other adverse conditions begin to appear. PWN stops breeding and starts to form the third-stage dispersal juvenile (DJ3). DJ3 accumulates a large number of small lipid droplets in the body, which is a long-lived stress-resistant stage, is supposed to play an important role in the northern expansion of this species, as it can survive under unfavorable conditions inside the deadwood of the host tree from autumn to the following spring [4–6]. Additionally, DJ3 is the prerequisite of the fourth-stage dispersal juvenile (DJ4), which can be transmitted with the insect vectors, *Monochamus* beetles [4, 7]. When the beetles feed on the branches of healthy pine trees, PWN enters the pine trees and begins its propagation cycle. Therefore, the control of DJ3 should allow for the prevention of the spread of this nematode species.

The complex life cycle of PWN is the result of the combination of environmental stimulation and gene expression change. DJ3 is morphologically and functionally similar to the dauer stage, which is a long-lived stress-resistant stage [8], of the model organism *Caenorhabditis elegans* [5, 8]. It has been reported that the biosynthesis of the disaccharide trehalose is involved in the regulation of dauer formation by interfering with dafachronic acid (DA) synthesis in *C. elegans* [9, 10]. Secreted DAs act to suppress the dauer-promoting activity of a nuclear hormone receptor/transcription factor, DAF-12 [11, 12], at the whole-organism level, and the worms remain in the reproductive cycle [13]. A cofactor for DA production is cytosolic NADPH, which is used by a cytochrome P450 (CYP450), DAF-9, as an electron donor [13]. *C. elegans* produces trehalose from glucose-6-phosphate (G6P) and uridine diphosphate glucose (UDPG) [14, 15]. G6P is an important molecule at the crossroads of several carbohydrate pathways. NADPH in the cytosol is predominantly produced from G6P in the oxidative phase of the pentose phosphate pathway (PPP) [16]. Therefore, trehalose biosynthesis and NADPH biosynthesis are coupled by their common substrate. The biosynthesis of trehalose diverts G6P from the PPP, which is the major source of NADPH in the cell. This results in a decrease in DA synthesis and an increase in dauer formation [9].

Trehalose is produced in a two-step reaction in *C. elegans* and most eukaryotes [14, 15]. First, trehalose-6-phosphate is synthesized by trehalose-6-phosphate synthase (TPS). Next, the phosphate is removed by trehalose-6-phosphate phosphatase (TPP). The hydrolysis of trehalose is catalyzed by trehalase (TRE). Trehalose has a protective effect on the survival of nematode in an adverse environment, and trehalose metabolic genes are involved in various stress resistance of nematode [17–19]. The biosynthesis of trehalose is induced by stress conditions such as cold, dehydration, oxidative stress, and so on [19–23]. The cold and dry climate in northern China may be a factor that induces the accumulation of trehalose and the formation of more DJ3. Trehalose also protects against stress in biological systems because it interacts with and directly protects lipid membranes and proteins from the damage caused by environmental stresses [20, 21]. Therefore, DJ3 in northern China may contain higher trehalose levels [24] and be more adaptable to an adverse environment, making it possible for the nematodes to survive and advance northward.

In addition to protecting the nematode against adverse conditions, metabolic shift from glycogen to trehalose promotes lifespan and healthspan in *C. elegans*, indicating the benefit of increasing trehalose [25]. Recent progress has demonstrated that trehalose is related to autophagy both in plants and animals [26–28]. Autophagy is an intracellular degradation system that delivers cytoplasmic constituents to the lysosome [29]. Autophagy can provide a quick supply of energy, or material to replace cell components, so it plays an essential role in the face of starvation and other kinds of stress. It has also been reported that autophagy is an important cellular pathway for dauer development and life-span extension in *C. elegans* dauer [30]. Therefore, trehalose metabolism may be associated with energy conversion related to autophagy, and may be involved in the regulation of DJ3 formation and low-temperature resistance. This study presented our identification and characterization of trehalose metabolism genes in PWN and our investigations on the role of trehalose metabolism in DJ3 formation and low-temperature survival.

## Results

### Digital gene expression (DGE) sequencing

To characterize the gene transcript patterns in different stages, 21 libraries (three replicates for each stage: second-stage propagative juvenile, J2; third-stage propagative juvenile, J3; fourth-stage propagative juvenile, J4; second-stage propagative juveniles prior to DJ3, J2-2; DJ3; female and male) of PWN were constructed and sequenced. After removing the low-quality reads, an average of 23.49 Mb clean reads were obtained for each sample (Additional file 1: Table S4). The datasets were deposited in the Sequence Read Archive (Accession: SRR10097294; BioProject ID: PRJNA564758). A total of 17,811 assembled unigenes were generated from the 21 libraries.

### Weighted correlation network analysis (WGCNA) revealed the module highly related to DJ3

**Fig. 2:**
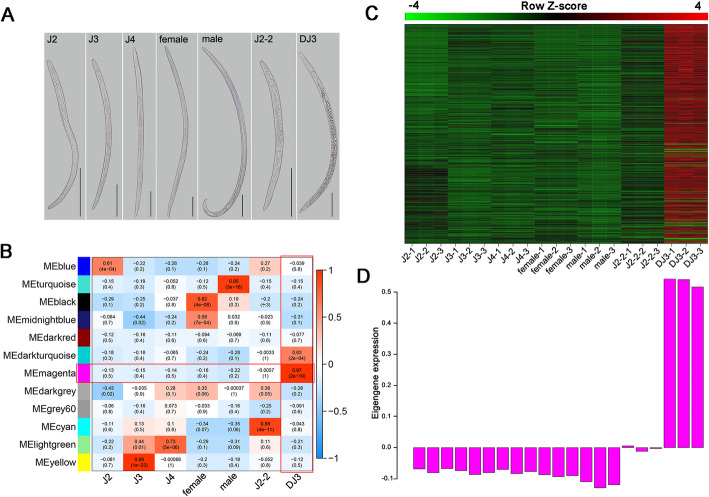
WGCNA revealed gene-network modules enriched in DJ3. **a.** Different developmental stages of pine wood nematodes. Bars indicate 100 *μ*m. **b.**Relationship between module and trait. **c.** Expanded view of the expression of all genes in the magenta module across all 21 samples. **d.**Eigengene expression for genes in the magenta module

To eliminate noise from genes that were not expressed, probes with median fragments per kilobase per million mapped fragments (FPKM) levels that did not exceed 1.0 were filtered. To explore the pathways related to DJ3, coexpression modules using the expression data of 15,899 genes (mean FPKM ≥ 1) from 21 libraries of PWN from different PWN stages (Fig. 2 A) were constructed by applying WGCNA. The stages to which the nematodes belonged were selected as traits. A power of 8 was chosen to construct the coexpression modules, and 12 gene-coexpression modules were identified in total (Additional file 1: Figure S1). Modules with common expression pattern interactions in the coexpression modules that were associated with particular traits were identified based on the correlation between the module eigengene (ME) and trait. The ME is defined as the first principal component of a given module. It can be considered a representative of the gene expression profiles in a module. The analysis identified that the magenta module was significantly associated with DJ3 (eigengene significance, ES = 0.97, *p* value = 2 × 10^− 19^, Fig. 2B). We decided to focus initially on the magenta module which had enriched expression in DJ3 (Fig. 2 C-D).

Kyoto Encyclopedia of Genes and Genomes (KEGG) enrichment analyses were performed on the genes in the magenta module. The top 20 pathways with the most enriched genes were shown in Additional file 1: Figure S2A. More than half (11) of these pathways belonged to metabolism (yellow-green), 4 belonged to cellular processes (pink), and 2 belonged to organismal systems (orange). These pathways included metabolic pathways (ko01100), lysosome (ko04142), carbon metabolism (ko01200), apoptosis (ko04210), peroxisome (ko04146), autophagy – animal (ko04140), longevity regulating pathway – worm (ko04212), and insulin signaling pathway (ko04910). In general, most of the pathways were enriched in global and overview maps (belongs to metabolism), transport and catabolism (belongs to cellular processes), carbohydrate metabolism (belongs to metabolism), and lipid metabolism (belongs to metabolism, Additional file 1: Figure S2B).

Further analysis was carried out on carbohydrate metabolism and lipid metabolism (Additional file 1: Figure S2C-D) as DJ3 accumulates a large number of energy substances to adapt to and survive the long-term adverse environment, and its formation is closely related to energy metabolism and storage in the body. Between genes enriched in carbohydrate metabolism, 2 of them were enriched to starch and sucrose metabolism (ko00500) and were involved in trehalose metabolism (TPS, EC2.4.1.15; TPP, EC3.1.3.12; Additional file 1: Figure S2E). To explore the effect of trehalose on the formation of DJ3 and the resistance to low-temperature, trehalose metabolic genes were selected for further research.

### Identification of TPS- and TRE-encoding genes

Homologous encoding genes of TPS (*Bx-tps*), TPP (*Bx-tpp*) and TRE (*Bx-tre1*, *Bx-tre2*, *Bx-tre3*, *Bx-tre4*, *Bx-tre5*, *Bx-tre6* and *Bx-tre7*) from the PWN transcriptome were identified using BLASTX (Additional file 1: Table S5, NCBI Accession: MT094332 to MT094340). According to the result of transcriptome sequencing, the coding sequences were cloned and the conserved domain of the proteins encoded by the cloned genes were analyzed. The TPS encoded by *Bx-tps* is a hydrophilic protein located in the cytoplasm and has no signal peptide. The TPP encoded by *Bx-tpp* is a hydrophilic protein located in the cytoplasm and has no signal peptide. The TREs encoded by *Bx-tre1*, *Bx-tre2*, *Bx-tre3*, *Bx-tre4*, *Bx-tre5* and *Bx-tre6* are hydrophilic proteins located in the cytoplasm and have no signal peptide; these TREs are NTREs. The TRE encoded by *Bx-tre7* is a hydrophilic protein located in the cytoplasm and has a signal peptide; this TRE is an ATRE (Additional file 1: Figure S3).

### *Bx-tps* and *Bx-tpp* were highly associated with DJ3

**Fig. 3:**
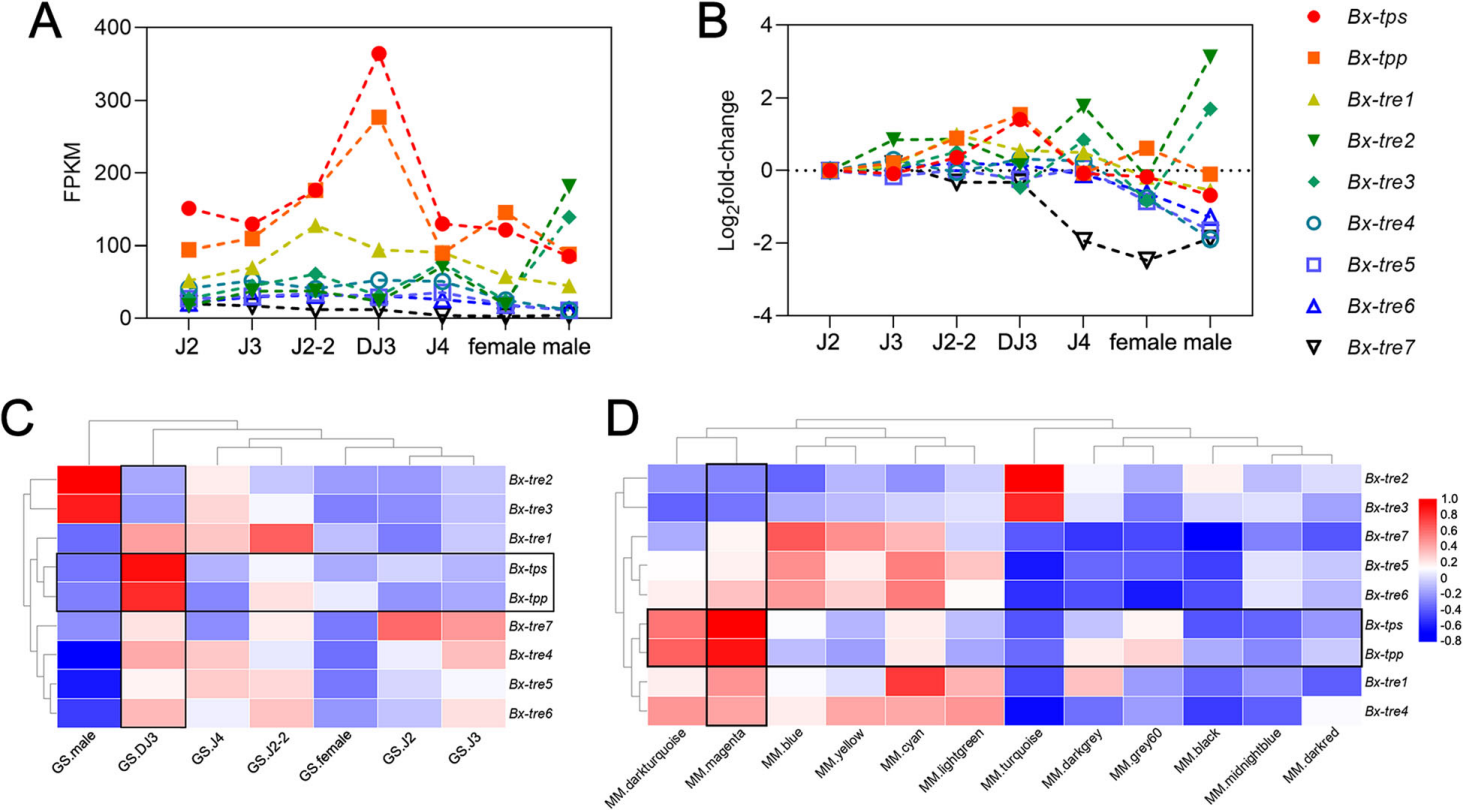
Trehalose metabolism is involved in the formation of DJ3. **a.** Trehalose metabolism gene expression patterns in different PWN stages based on FPKM. Data are given as the mean. **b.** Transcript relative abundance of trehalose metabolism genes. Data are given as the mean. **c.** The gene significance (GS) of trehalose metabolism gene expression at each PWN stage. **d.** The module membership (MM) of trehalose metabolism gene expression and each module eigengene (ME)

By comparing the expression levels of trehalose metabolism genes at different stages, it was found that the expression levels of *Bx-tps* and *Bx-tpp* were significantly higher in DJ3 than those in other stages (*p* value < 0.01, Fig. 3 A-B). The gene significance (GS) between the gene expression profile and each PWN stage indicated that the expression profiles of *Bx-tps* (correlation value, cor = 0.90, *p* value = 2.16 × 10^− 11^) and *Bx-tpp* (cor = 0.80, *p* value = 1.09 × 10^− 7^) were highly associated with the DJ3 stage (Fig. 3 C). The module membership (MM) between the gene expression profile and each ME indicated that the expression profile of the eigengene of the magenta module was highly associated with the expression profiles of *Bx-tps* (cor = 0.95, *p* value = 3.97 × 10^− 15^) and *Bx-tpp* (cor = 0.89, *p* value = 4.95 × 10^− 11^, Fig. 3D).

### Trehalose metabolism is essential for the formation of DJ3

**Fig. 4:**
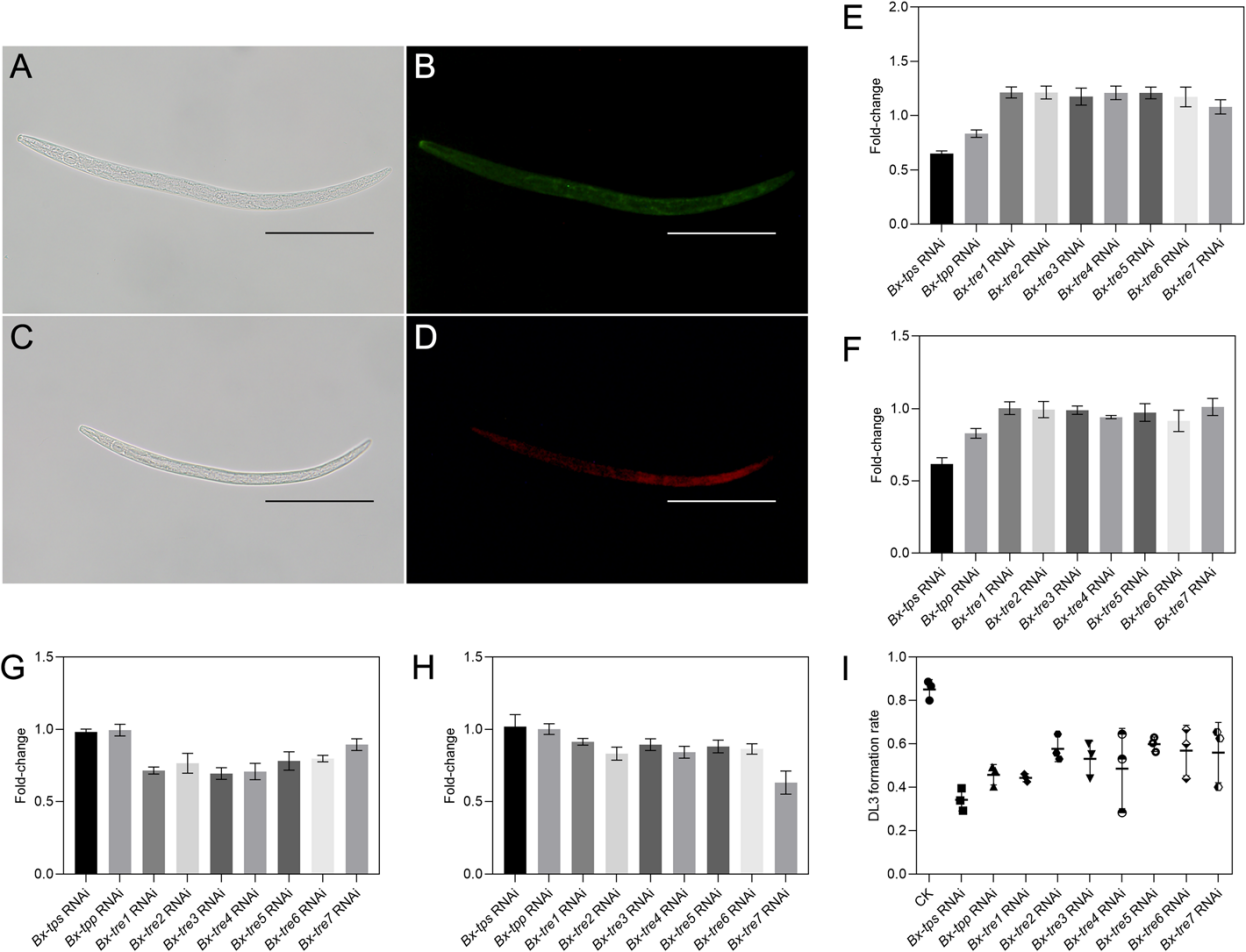
J2-2 treated with siRNA and the trehalose level, TPS activity, TRE activity, and DJ3 formation rate after RNAi. **a** RNAi-treated J2-2. **b** Green fluorescence indicates FAM-labeled siRNA corresponding to effective siRNA entry into J2-2. **c** Control check J2-2. **d** Red fluorescence indicates Cy3-labeled nt siRNA corresponding to effective siRNA entry into J2-2. **e** Fold-changes of trehalose level after RNAi treatment. **f** Fold-changes of TPS activity after RNAi treatment. **g** Fold-changes of NTRE activity after RNAi treatment. **h** Fold-changes of ATRE activity after RNAi treatment. **i** DJ3 formation rate changes. Bars indicate 100 *µ*m. Data are given as the mean with s.d. (*n* = 3)

Potent and specific silencing was found in J2-2 after treatment with the matching siRNA for 12 h. The observation of fluorescein indicated that FAM/Cy3-labeled siRNA was effectively taken up by RNAi-treated J2-2 (Fig. 4 A-D). RT-qPCR showed significant suppression of matching trehalose metabolism genes (Additional file 1: Table S6, *p* value < 0.01). As a result of knocking down *Bx-tps* or *Bx-tpp*, the trehalose level and TPS activity decreased (*p* value < 0.01, Fig. 4E, F). After knocking down *Bx-tre1*, *Bx-tre2*, *Bx-tre3*, *Bx-tre4*, *Bx-tre5* or *Bx-tre6* the trehalose level increased, while NTRE activity decreased (*p* value < 0.01, Fig. 4E, G). After knocking down *Bx-tre7*, the trehalose level increased, while ATRE activity decreased (*p* value < 0.01, Fig. 4E, H). The formation rate of DJ3 decreased after RNAi treatment for each trehalose metabolism gene with *Bx-tps* RNAi treatment decreased the most (*p* value < 0.01, Fig. 4I).

### Cold treatment induced trehalose accumulation in DJ3

**Fig. 5:**
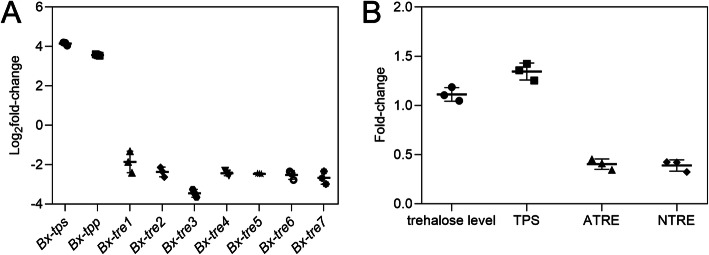
Changes of transcript abundance, trehalose level, TPS activity and TRE activity of cold-treated DJ3. **a** Changes of transcript abundance of trehalose metabolism genes of cold-treated DJ3. **b** Changes of trehalose level, TPS activity, ATRE activity and NTRE activity of cold-treated DJ3. Data are given as the mean with s.d. (*n* = 3)

We compared the gene expression patterns of trehalose metabolism-related genes, trehalose levels, TPS activity and TRE activity of cold-treated DJ3 and control check DJ3 (named CK2). The expression levels of *Bx-tps* and *Bx-tpp* in cold-treated DJ3 were significantly higher than those in CK2 (*p* value < 0.01), while the expression levels of *Bx-tre1, Bx-tre2, Bx-tre3, Bx-tre4, Bx-tre5, Bx-tre6* and *Bx-tre7* were significantly lower than those in CK2 (*p* value < 0.01, Fig. 5 A). The trehalose level and TPS activity of cold-treated DJ3 were much higher than that of CK2 (*p* value < 0.01), while the TRE activity of cold-treated DJ3s was much lower than that of CK2 (*p* value < 0.01, Fig. 5B). These results indicated that a low-temperature like − 20 °C induced the nematode to accumulate trehalose.

### Trehalose is essential for frigid-temperature survival of DJ3

**Fig. 6:**
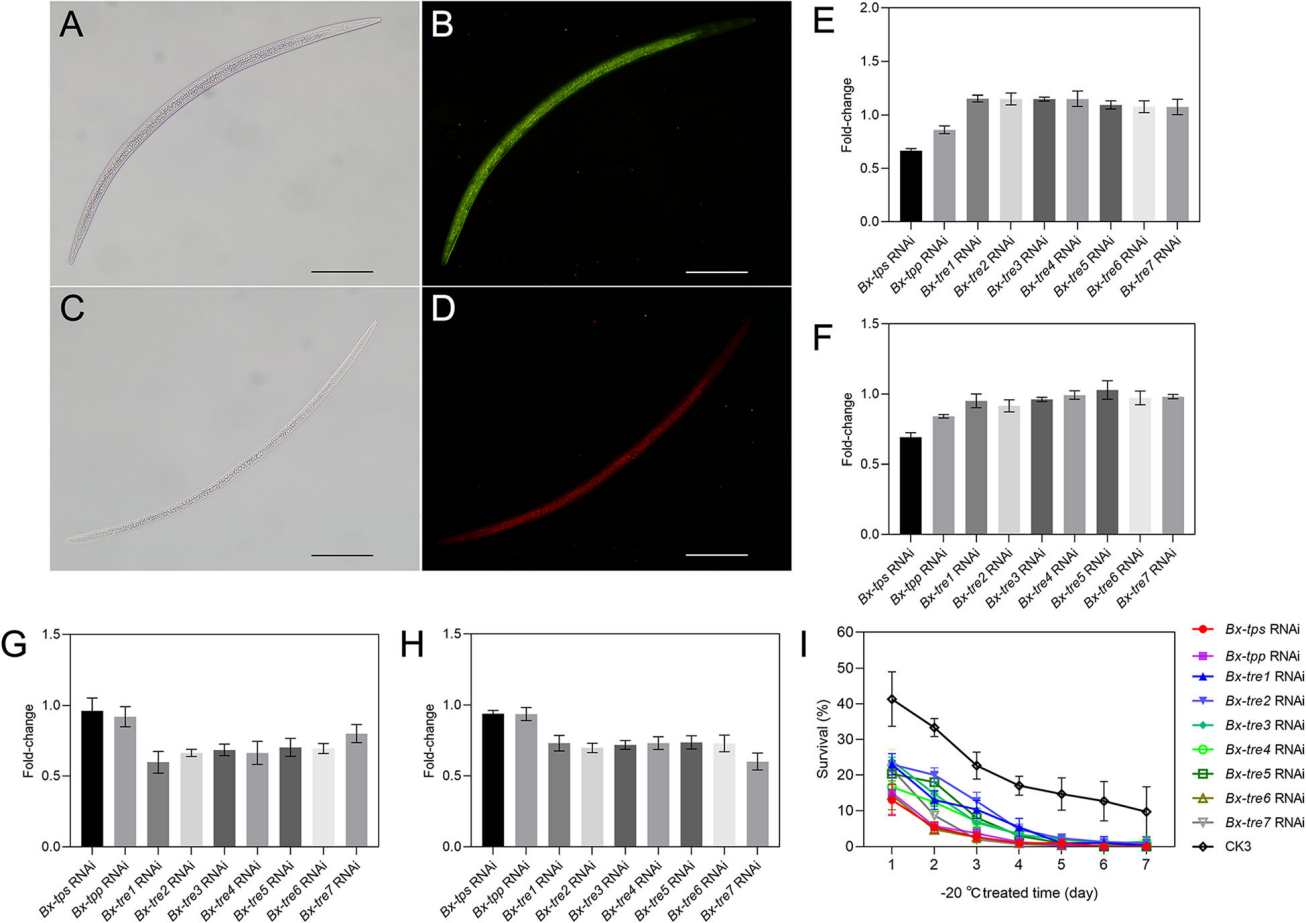
DJ3 treated with siRNA and the trehalose level, TPS activity, TRE activity, and low-temperature survival rate after RNAi. **a** RNAi-treated DJ3. **b** Green fluorescence indicates FAM-labeled siRNA corresponding to effective siRNA entry into DJ3. **c** Control check DJ3. **d** Red fluorescence indicates Cy3-labeled nt siRNA corresponding to effective siRNA entry into DJ3. **e** Fold-changes of trehalose level after RNAi treatment. **f** Fold-changes of TPS activity after RNAi treatment. **g** Fold-changes of NTRE activity after RNAi treatment. **h** Fold-changes of ATRE activity after RNAi treatment. **i** Survival at -20 °C. Bars indicate 100 *µ*m. Data are given as the mean with s.d. (*n* = 3)

Potent and specific silencing was found in DJ3 after treatment with the matching siRNA for 12 h. The observation of fluorescein indicated that FAM/Cy3-labeled siRNA was effectively taken up by RNAi-treated DJ3 (Fig. 6 A-D). RT-qPCR showed significant suppression of matching trehalose metabolism genes (Additional file 1: Table S7, *p* value < 0.01). As a result of knocking down *Bx-tps* or *Bx-tpp*, the trehalose level and TPS activity decreased (*p* value < 0.01, Fig. 6E, F). After knocking down *Bx-tre1, Bx-tre2, Bx-tre3, Bx-tre4, Bx-tre5* or *Bx-tre6*, the trehalose level increased, while NTRE activity decreased (*p* value < 0.01, Fig. 6E, G). After knocking down *Bx-tre7*, the trehalose level increased, while ATRE activity decreased (*p* value < 0.01, Fig. 6E, H).

There was no distinct difference in survival rate and morphology between RNAi-treated DJ3 and control check DJ3 (named CK3) when they were inoculated in Japanese black pines (*P. thunbergii*) and cultured at room temperature. The survival rate decreased at -20 °C after RNAi treatment and the survival rate of DJ3 was the lowest after *Bx-tps* silencing (*p* value < 0.05, Fig. 6I). The above results indicated that the accumulation of trehalose was beneficial for DJ3 survival under a frigid temperature of -20 °C and trehalose metabolism genes might need to work together to help the survival of DJ3 at -20 °C.

### *Bx-tps* and *Bx-tpp* coexpressed genes are enriched in metabolic related pathways

**Fig. 7:**
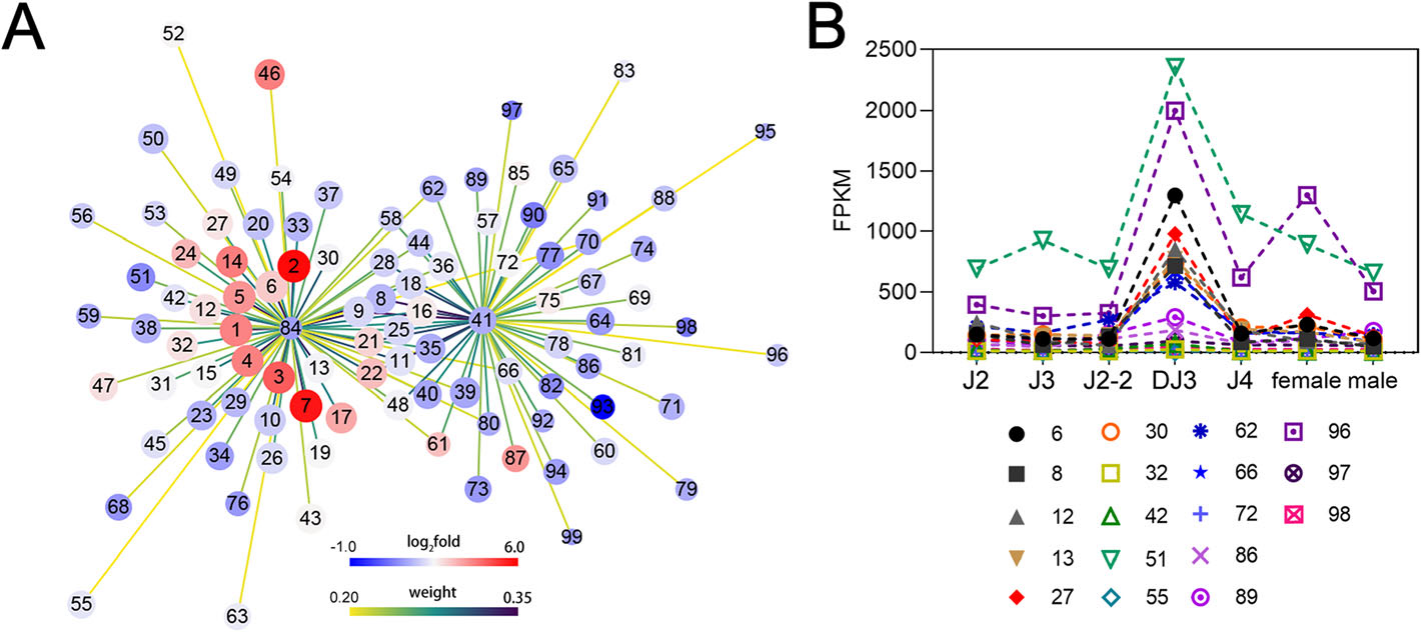
*Bx-tps* and *Bx-tpp* coexpressed genes. **a.** Gene coexpression network for *Bx-tps* and* Bx-tpp* in the pink module (weight value>0.2, detailed in Additional file 1: Table S8). Prefuse Force Directed Layout was applied based on the weight value between two genes. The size of the dots represents the GS (from 0.6757 to 0.9984). The color of the dots represents log_2_fold change (from 1.0 to 6.0) for the FPKM of DJ3 vs. the average FPKM of the other stages of PWN. The colors of the lines represent the weight value between two genes (from 0.20 to 0.35). The labels on the dots are based on intramodular connectivity (IC, from 32.86 to 190.43). No. 41 and No. 84 represent* Bx-tps* and *Bx-tpp*, respectively. **b.** The expression patterns of selected genes in different stages of PWN. Data are given as the mean

Based on WGCNA results, genes highly correlated with *Bx-tps* and *Bx-tpp* in the magenta module were screened. The genes in the magenta module whose weight values with *Bx-tps* or *Bx-tpp* were greater than 0.2 were selected, and the network data were exported to Cytoscape by Prefuse Force Directed Layout based on the weight value between two genes (Fig. 7 A, Additional file 1: Table S8). The whole network contained 119 regulatory relationships of *Bx-tps* and *Bx-tpp* with 98 genes and *Bx-tps* and *Bx-tpp* may regulate each other. Among those 98 genes in the magenta module that had a high weight value with *Bx-tps* or *Bx-tpp*, 56 of them were enriched in 72 different pathways (Additional file 1: Figure S4A). Among these genes, 9 were enriched in lipid metabolism (ko00061, ko00561, ko00564, ko00062, ko00071 and ko01040); 9 were enriched in carbohydrate metabolism (ko00620, ko00640, ko00010, ko00630, ko00020, ko00030 and ko00500); 7 were enriched in signal transduction (ko04152, ko04013, ko04072, ko04150, ko04310 and ko04390); 7 were enriched in endocrine system (ko04910, ko04922, ko04916, ko04919, ko04925 and ko04928) and 6 were enriched in digestive system (ko04975, ko04977, ko04974 and ko04979).

The KEGG pathways enriched by more genes mostly belonged to metabolism (Additional file 1: Figure S4B, green bars). Between them, pyruvate metabolism (ko00620), propanoate metabolism (ko00640), carbon metabolism (ko01200), fatty acid metabolism (ko01212), glycolysis/gluconeogenesis (ko00010), fatty acid biosynthesis (ko00061), glycerolipid metabolism (ko00561), glyoxylate and dicarboxylate metabolism (ko00630), glycerophospholipid metabolism (ko00564) and fat digestion and absorption (ko04975) were enriched by more genes. Besides, many genes enriched in AMPK signaling pathway (ko04152), insulin signaling pathway (ko04910) and glucagon signaling pathway (ko04922), which belong to environmental information processing (Additional file 1: Figure S4B, blue bars). And lysosome (ko04142) and peroxisome (ko04146) in the cellular processes (Additional file 1: Figure S4B, pink bars) also had relatively more genes enriched in.

The above 15 pathways indicated that most genes in the gene coexpression network were involved in metabolic processes, including glucose metabolism, carbon metabolism and fatty acid metabolism, and were associated with multiple longevity related signaling pathways. A total of 18 genes were enriched in the above 15 pathways, among which several genes were enriched in multiple pathways (Additional file 1: Table S9). Genes were numbered from large to small based on intramodular connectivity (IC) values. Genes No. 42, No. 30 and No. 13 enriched in 7 pathways respectively, including related pathways of carbohydrate metabolism, fat metabolism, signal transduction and endocrine system. In addition, genes No. 6 and No.32 enriched in 6 pathways, all of which were associated with carbohydrate metabolism. The annotated results of the 18 genes in the nr library were shown in Additional file 1: Table S10. The expression levels of these genes in DJ3 were significantly higher than those in other PWN stages (Fig. 7B), indicating that these genes may play an important role in DJ3. Some of the genes had high weight values with *Bx-tps* or *Bx-tpp*, suggesting that these metabolic-related genes may have a regulatory relationship with trehalose metabolism genes and participate in the maintaining of DJ3.

## Discussion

Trehalose is a multifunctional nonreducing disaccharide that exists naturally in all kingdoms [20]. Trehalose can protect cells and proteins from extreme environmental damage and provide energy and carbon sources as well [20]; in addition, trehalose aids organisms in resisting adverse conditions. Diverse environmental stresses may result in different expression levels of trehalose metabolism genes [22, 23]. Once the body senses an external signal, the mechanisms involved in trehalose metabolism are triggered, and metabolic pathways are regulated [21–23, 31]. Therefore, trehalose is often used as an effective cytoprotective agent under extreme temperature, radiation and dehydration in many organisms [32, 33].

In this study, we found that the expression of trehalose metabolism genes was related to the formation of DJ3 and the formation rate of DJ3 was the lowest after *Bx-tps* silencing. Trehalase encoding gene silencing may produce negative feedback for the synthesis of trehalose, as the organisms need to maintain a balance of trehalose in their bodies [9, 22, 23]. Therefore, reducing the expression of the trehalase gene may eventually lead to a decrease in trehalose synthesis. This result indicated that some environmental stress stimuli that promote trehalose accumulation may also promote DJ3 formation [9].

Low-temperature stimulation can usually induce trehalose accumulation. After cold treatment, the expression levels of *Bx-tps* and *Bx-tpp* were upregulated, and the trehalose level increased, while the expression levels of *Bx-tre*s were downregulated and TRE activity decreased, indicating that a low-temperature, such as -20 °C, may induce trehalose accumulation. This suggested that DJ3 may accumulate trehalose when sensing low-temperatures to enable survival. By gene silencing of trehalose metabolism genes, we found that a decrease in trehalose levels reduced the survival rate of DJ3 at -20 °C. But an increase in trehalose levels and a decrease in TRE activity also reduced the survival rate of DJ3 at-20 °C. These results suggested that trehalose accumulation is beneficial for DJ3 to survive in pine trees at a frigid temperature such as -20 °C, but trehalose may also need to provide energy and carbon as well as protect cells and proteins to enable DJ3 survival at -20 °C. This also means trehalose metabolism genes need to work together to confers resistance to low-temperature stress.

These results suggested that low-temperatures may result in the presence of more DJ3 by increasing the conversion rate and survival of DJ3 [9, 32, 34, 35]. PWN usually occurs in hot and dry climates. Low-temperatures during winter in the northeast areas in China present a serious challenge to its colonization of this region. As a long-lived stress-resistant stage, DJ3 can survive under a variety of environmental stresses. This ability may in part be due to low-temperature stimulation promoting the accumulation of trehalose in this nematode [24, 32, 34, 36]. Furthermore, the synthesis of trehalose will promote the formation of DJ3 [9], and as a result, the nematode may gradually be able to adapt to survival at increasingly lower temperatures and gradually spread to cold northern areas. Therefore, it is essential to understand the mechanisms of trehalose in this nematode.

Through WGCNA, we found that *Bx-tps* and *Bx-tpp* were strongly correlated with the DJ3 stage. This indicates that, compared to the hydrolysis of trehalose, the synthesis of trehalose may play a more important role in the formation and maintaining of DJ3. Further analysis showed that many of the genes highly associated with DJ3 and with *Bx-tps* and *Bx-tpp* were involved in metabolic processes, including glucose metabolism, carbon metabolism and fatty acid metabolism, and were associated with multiple longevity-related signaling pathways. In addition, many genes enriched in AMPK signaling pathway (ko04152), insulin signaling pathway (ko04910) and glucagon signaling pathway (ko04922). And lysosome (ko04142) and peroxisome (ko04146) also had relatively more genes enriched in. These pathways are thought to be associated with energy conversion and autophagy.

Recently, trehalose has been implicated in autophagy [25, 37]. Autophagy is associated with extended lifespan in mammals and plants due to its homeostatic role of removing damaged, unwanted cellular components as well as cellular toxins [29, 38, 39]. A growing body of evidence suggests that autophagy pathways play a dual role in promoting both cell survival and cell death. In plants, the accumulation of trehalose may maintain the efficiency of autophagy pathways when it is most needed [26]. Trehalose was reported to inhibit members of the solute carrier 2 A (SLC2A) family (also referenced as the glucose transporter, or GLUT family) of glucose transporters and trehalose-mediated inhibition of glucose transport induced AMPK (adenosine 5′-monophosphate-activated protein kinase)-dependent autophagy regression of hepatic steatosis in vivo [27]. It has also been reported that autophagy is a cellular pathway essential for dauer development and lifespan extension in *C. elegans* [30]. In this study, we found 18 genes involved in energy conversion and autophagy highly related to *Bx-tps* and *Bx-tpp* and to DJ3. *Bx-tps* and *Bx-tpp* may be involved in autophagy by regulating trehalose level and affecting expression levels of these genes. These energy conversions and autophagy-related genes may also play important roles in DJ3 formation and maintaining as they highly expressed in DJ3. However, how *Bx-tps*, *Bx-tpp* and these genes regulate each other requires further verification.

## Conclusions

The expression of trehalose metabolism genes is related to the formation of DJ3. The accumulation of trehalose is beneficial for DJ3 in response to low-temperature stress and trehalose metabolism genes need to work together to confers resistance to low-temperature stress. *Bx-tps* and *Bx-tpp* are highly correlated with DJ3 and may be involved in autophagy by regulating energy conversion related genes. These energy conversion and autophagy-related genes may also play important roles in DJ3 formation and maintaining. Inhibition of the expression of these genes may reduce the formation of DJ3 or the survival rate of DJ3 at low temperatures, so as to reduce the probability of nematode proliferation and prevent nematode from further spreading to low temperatures.

## Methods

### cDNA library construction and DGE sequencing

The test nematodes were collected from Dalian, Liaoning Province, China (121°33’9.234’’ east longitude and 38°54’26.892’’ north latitude) in April 2017 and cultured on *Botrytis cinereal* at 25 °C in the lab. Artificially induced DJ3 was cultured according to the method of Ishibashi and Kondo with a few modifications [40]. Different stages of propagative juveniles were obtained by culture on *B. cinerea* and placed in a 25 °C incubator for certain periods. The developmental stages of nematodes were distinguished under a microscope. J2-2 was collected before the nematode turned into DJ3. Total RNA for 21 groups of nematodes from different stages (three replicates for each stage: J2, J3, J4, J2-2, DJ3, female and male) was extracted using TRIzol (Invitrogen, USA, cat. no. 15596-026) [41]. For each replicate, about 10,000 nematodes for each stage were used for RNA extraction except for J2 or J2-2 which used about 30,000 nematodes. The concentration, RNA integrity number (RIN), 28 S/18S and fragment size of the total RNA were examined by the RNA Nano 6000 Assay Kit of the Agilent 2100 Bioanalyzer system (Agilent, USA). A NanoDrop™ (Thermo Scientific, USA) was used to examine the purity of the RNA. Amplification grade DNase I (Invitrogen, USA, catalogue number: 18068-015) was used to remove genomic DNA.

Each RNA was sheared and reverse transcribed using random primers to obtain cDNA for library construction. The construction of libraries and sequencing were all performed on a BGISEQ-500 RNA-seq platform (BGI, Shenzhen, China), and 50-bp single-end (SE) reads were generated. The raw sequencing reads generated were filtered by SOAPnuke (v1.5.2, https://github.com/BGI-flexlab/SOAPnuke). HISAT2 (v2.0.4, http://www.ccb.jhu.edu/software/hisat) was used to map the clean reads to the reference genome of PWN (BioSample: SAMEA2272519, http://www.ncbi.nlm.nih.gov/assembly/310678) [42]. Bowite2 was used (v2.2.5, http://bowtie-bio.sourceforge.net/Bowtie/index.shtml) to map clean reads to reference sequences [43]. RSEM (v1.2.12, http://deweylab.biostat.wisc.edu/RSEM) was used to calculate and normalize the matched reads to the FPKM values [44].

### WGCNA

WGCNA was used to explore the complex relationships between genes and phenotypes (https://horvath.genetics.ucla.edu/html/CoexpressionNetwork/) [45]. The appropriate power value was determined when the degree of independence was over 0.8. The minimum number of genes was set as 30 for the high reliability of the results. Module-trait associations were estimated using the correlation between the module eigengene (ME) and the trait. The intramodular connectivity (IC) was calculated for each gene by summing the connection strengths with those of other module genes and dividing this number by the maximum intramodular connectivity. For each expression profile, gene significance (GS) was calculated as the absolute value of the Pearson correlation between the expression profile and each trait. Module membership (MM) was defined as the Pearson correlation of the expression profile and each ME. Network depictions were constructed with Cytoscape software [46]. KEGG [47] enrichment analysis was performed on selected genes. The results of the analyses were extracted, and a *p* value ≤ 0.05 after the correction was used as the threshold.

### Identification of homologous trehalose metabolism genes

A Baermann funnel was used to extract the nematodes (male, female, and juvenile mixed at the ratio of 1:1:2). About 20,000 nematodes were collected and then frozen with liquid nitrogen and powdered. Total RNA for PWN was extracted using TRIzol [41]. The Promega AMV reverse transcription system was used (Promega, USA, cat. no. A3500) to obtain the first chain of cDNA.

One putative TPS-encoding genes, one putative TPP-encoding gene and five putative TRE-encoding genes have been identified in *C. elegans* [14, 15]. Homologous TPS- (*Bx-tps*), TPP- (*Bx-tpp*) and TRE-encoding genes (*Bx-tre*) from the PWN transcriptome (BioSample: SAMEA2272519, http://www.ncbi.nlm.nih.gov/assembly/310678) were identified using BLASTX and their encoding sequences were cloned using cDNA as templates. The primers used here are listed in Additional file 1: Table S1. Trehalose hydrolysis is catalyzed by two types of TRE, acid TRE (ATRE) [48] and neutral TRE (NTRE) [49]. ATRE and NTRE are responsible for the utilization of extracellular trehalose and the mobilization of intracellular trehalose, respectively [49, 50]. To identify the type of TRE (NTRE or ATRE), SignalP4 (http://www.cbs.dtu.dk/services/SignalP/) was used to identify signal peptides; ScanProsite (http://prosite.expasy.org/) was used to analyze structural characteristics of proteins, and PSORTII Prediction (http://psort.hgc.jp/form2.html) was used to predict the subcellular localizations [22].

### Determination of TRE activity and trehalose level

For each treatment, about 30,000 nematodes were collected for the determination of TRE activity and trehalose level. TRE activity was determined with the TRE Determination Kit (acidic version/neutral version; Cominbio, China, catalogue number: HTM-2-Y) and the BCA Method of Protein Content Kit (Cominbio, catalogue number: BCAP-2-W) as described before [22, 48]. TPS activity was determined by using Trehalose-6-Phosphate Synthase (TPS) Kit (Geruisi, China, catalogue number: G0556F). Trehalose levels were measured with the Trehalose Content Kit (Cominbio, catalogue number: HT-2-Y) as described before [22].

For each test, the color depth of the reaction fluids was measured by a GeneQuant 1300 ultraviolet spectrophotometer (Biochrom Ltd., UK). Three separate biological replicates of each treatment were performed, and each replicate was assessed three times. Student’s *t*-test was used to determine the significance.

### Analysis of trehalose participation in DJ3 formation

J2-2 were collected to perform RNAi. Fluorescent siRNAs labeled with 5-carboxyfluorescein (FAM) corresponding to *Bx-tps*, *Bx-tpp*, *Bx-tre1*, *Bx-tre2*, *Bx-tre3*, *Bx-tre4*, *Bx-tre5*, *Bx-tre6* or *Bx-tre7* were constructed separately. As a nontargeting RNAi treatment, a random sequence was selected as nontargeting siRNA (nt siRNA) and labeled with cyanine dyes 3 (Cy3). The target sequence used here are listed in Additional file 1: Table S2.

The nematodes were treated at 20 °C with M9 buffer with 10 mmol l^− 1^ octopamine and the matching siRNA (2 mg ml^− 1^) for RNAi treatment or treated with the same amount of M9 buffer with 10 mmol l^− 1^ octopamine and nt siRNA (2 mg ml^− 1^) for the control check and named CK1 [22]. SiRNA marker FAM was detected under blue light and siRNA marker Cy3 was detected under green light to determine whether siRNA entered the nematode. The extent of RNAi was determined by measuring the transcript levels of trehalose metabolism genes, the trehalose level, the TPS activity and the TRE activity after treatment with the matching siRNA for 12 h. The nematodes were then cultured based on the method of Ishibashi and Kondo [31] for 14 d before being collected to calculate the rate of DJ3 formation. For each treatment, about 100,000 nematodes were used. Three separate biological replicates of each treatment were performed, and each replicate was assessed three times. Significance was determined by Student’s *t*-test.

### RT-PCR

The total RNA of differently treated nematodes was extracted from the powder using TRIzol reagent [41]. The transcript levels of trehalose metabolism genes were then measured by RT-qPCR using the GoTaq 2-Step RT-qPCR System Kit (Promega, USA, catalogue number: A6010) and a Stratagene Mx3000P qPCR system (Agilent, USA) according to the manufacturer’s instructions. The primers used here are listed in Additional file 1: Table S3. The RT-qPCR results were normalized as log_2_fold changes with a constitutively expressed gene, 28 S RNA, as an internal control. The ${2}^{-{\Delta }{\Delta }{C}_{T}}$ method was used to analyze the data [51]. Three separate biological replicates of each treatment were performed, and each replicate was assessed three times. Significance was determined by Student’s *t*-test.

### The effects of trehalose on PWN survival at low-temperature

DJ3 was cultured at -20 °C for one day for cold treatment or at 25 °C for the same time for the control check (named CK2). The transcript levels of trehalose metabolism genes, the trehalose level, the TPS activity and TRE activity for cold-treated DJ3 and CK2 were determined as described before. To investigate the participation of trehalose in low-temperature survival, RNAi treatments of each trehalose metabolism gene was performed on DJ3. DJ3 was soaked with M9 buffer with 10 mmol l^− 1^ octopamine and the matching siRNA for 12 h, respectively, for the RNAi treatments. The control check DJ3 (named CK3) was soaked with M9 buffer with 10 mmol l^− 1^ octopamine and nt siRNA (2 mg ml^− 1^) for the same time. For each treatment, about 120,000 nematodes were used for each treatment. The extent of RNAi was determined as described above.

Three-year-old Japanese black pines were provided by Liaoning Provincial Key Laboratory of Dangerous Forest Pest Management and Control, Shenyang, Liaoning Province, China. No permission was required to obtain these plant samples. To determine the survival changes, annual shoots of three-year-old Japanese black pines were inoculated with 5,000 differently treated DJ3 and the inoculated pines were put in incubators at -20 °C for 1 day to 6 days or put in incubators at the room temperature for 1 day to 6 days, respectively [52]. The survival of the DJ3 was monitored daily. Three separate biological replicates of each treatment were performed, and each replicate was assessed three times. Student’s *t*-test was used to determine the significance.

### Availability of supporting data

All supporting data are included as additional files as “Additional file 1.docx”.

## Supplementary Information

**Additional file 1: Figure S1.** Cluster Dendrogram. **Figure S2.**KEGG enrichment analyses of genes in the magenta module. **Figure S3.**Signal peptide analysis. **Figure S4.** KEGG enrichment analyses of *Bx-tps*and *Bx-tpp* coexpressed genes. **Table S1.** Primers used in cloning encoding sequence. **Table S2.**Target sequences for siRNA. **Table S3.**Primers used in RT-qPCR. **Table S4.** Statistical analysis of the RNA sequencing data. **Table S5.** Alignment results of trehalose metabolism related genes in pine wood nematode. **Table S6.** Expression of trehalose metabolism related genes after RNAi treatment. **Table S7.** Expression of trehalose metabolism related genes after RNAi treatment. **Table S8.** Selected genes in the magenta module with high weight value (>0.2) to *Bx-tps* or *Bx-tpp*.**Table S9.** KEGG enrichment for genes highly related to *Bx-tps1* and*Bx-tps2*. **Table S10.** Gene annotation result.

## Data Availability

The datasets are available in the Sequence Read Archive (Accession: SRR10097294; BioProject ID: PRJNA564758).

## Abbreviations

ATRE: acid TRE
CK: control check
Cy3: cyanine dyes 3
CYP450: cytochrome P450
DA: dafachronic acid
DGE: digital gene expression
DJ3: third-stage dispersal juvenile
DJ4: fourth-stage dispersal juvenile
FAM: 5-carboxyfluorescein
FDR: false discovery rate
FPKM: fragments per kilobase per million mapped fragments
G6P: glucose-6-phosphate
GS: gene significance
IC: intramodular connectivity
KEGG: Kyoto Encyclopedia of Genes and Genomes
J2: second-stage propagative juvenile
J2-2: second-stage propagative juveniles prior to DJ3
J3: third-stage propagative juvenile
J4: fourth-stage propagative juvenile
ME: module eigengene
MM: module membership
Nr: NCBI Nonredundant sequences database
NTRE: neutral TRE
PPP: pentose phosphate pathway
PWD: pine wilt disease
PWN: pine wood nematode
RIN: RNA integrity number
RT-qPCR: real-time quantitative PCR
SE: single-end
SRA: Sequence Read Archive
TPP: trehalose-6-phosphate phosphatase
TPS: trehalose-6-phosphate synthase
TRE: trehalase
Tween-20: polysorbate surfactant 20
WGCNA: weighted correlation network analysis
