# Supplementary material for: Trehalose in pine wood nematode participates in DJ3 formation and confers resistance to low-temperature stress
Source: BMC Genomics. 2021 Jul 9;22:524. doi: 10.1186/s12864-021-07839-0 (PMC8268229; doi:10.1186/s12864-021-07839-0)
Supplement: Supplementary file 1 — Additional file 1: Figure S1. Cluster Dendrogram. Figure S2.KEGG enrichment analyses of genes in the magenta module. Figure S3.Signal peptide analysis. Figure S4. KEGG enrichment analyses of Bx-tpsand Bx-tpp coexpressed genes. Table S1. Primers used in cloning encoding sequence. Table S2.Target sequences for siRNA. Table S3.Primers used in RT-qPCR. Table S4. Statistical analysis of the RNA sequencing data. Table S5. Alignment results of trehalose metabolism related genes in pine wood nematode. Table S6. Expression of trehalose metabolism related genes after RNAi treatment. Table S7. Expression of trehalose metabolism related genes after RNAi treatment. Table S8. Selected genes in the magenta module with high weight value (>0.2) to Bx-tps or Bx-tpp.Table S9. KEGG enrichment for genes highly related to Bx-tps1 andBx-tps2. Table S10. Gene annotation result. [file 12864_2021_7839_MOESM1_ESM.docx]

Trehalose in pine wood nematode participates in DJ3 formation and confers resistance to low-temperature stress

Qiaoli Chen^1,2§^, Ruizhi Zhang^1§^, Danlei Li^1,2^, Feng Wang^1,2*^, Shengwei Jiang^3§^, Jianan Wang^1^

^1^Key Laboratory of Detection and Control of Alien Forest Pests-Heilongjiang Province, School of Forestry, Northeast Forestry University, Harbin 150040, Heilongjiang, P. R. China; ^2^Key Laboratory of Sustainable Forest Ecosystem Management-Ministry of Education, Northeast Forestry University, Harbin 150040, Heilongjiang, P. R. China; ^3^Station of Forest and Grassland Pest Control and Quarantine, Shenyang, Liaoning 110001, P. R. China

^*^Corresponding author.

^§^These authors contributed equally to this work.

E-mail addresses: qiaolichen@nefu.edu.cn (Q. Chen), zhangruizhi@nefu.edu.cn (R. Zhang), danleili@nefu.edu.cn (D. Li), kingsummit@126.com (F. Wang), jiangshengwei@iae.ac.cn (S. Jiang), wangjianan@nefu.edu.cn (J. Wang).


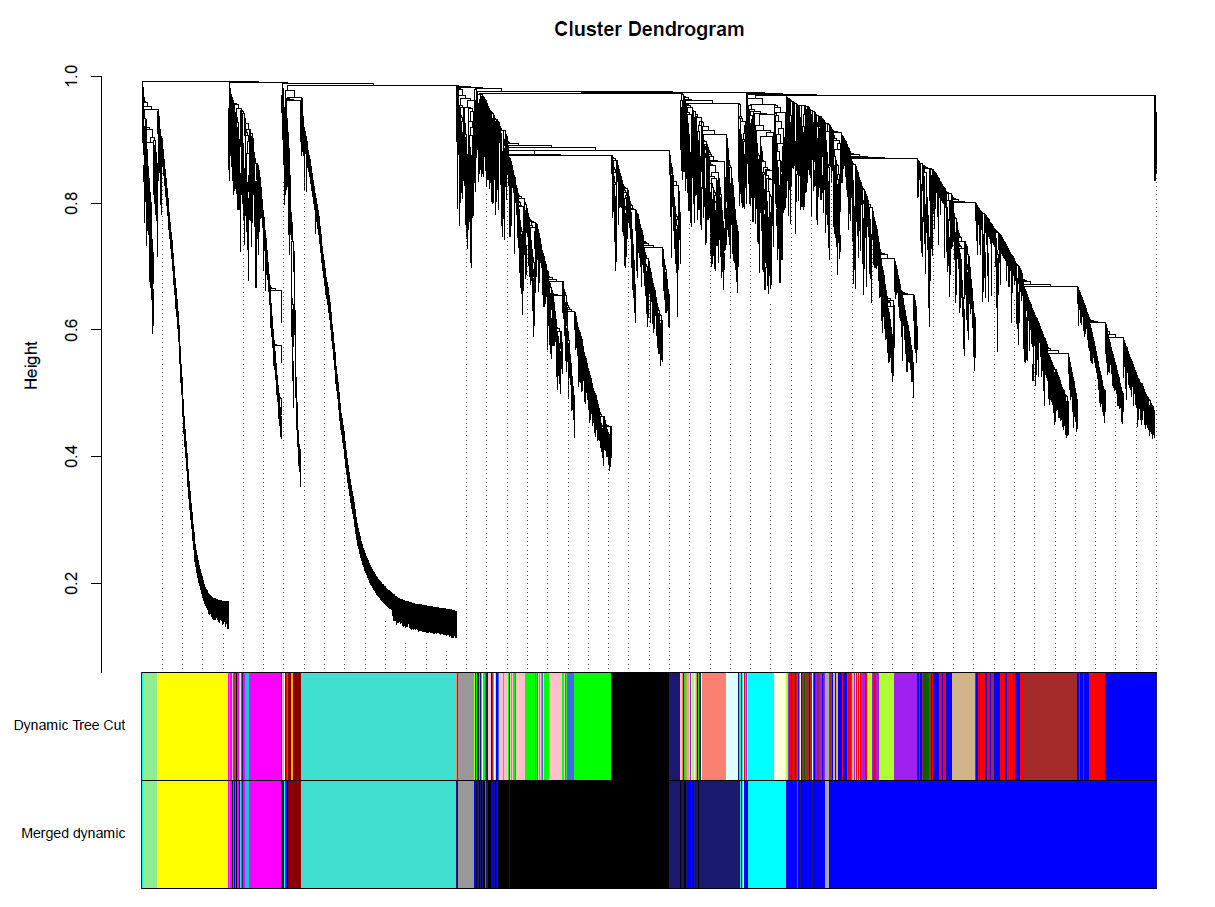


Figure S1. Cluster Dendrogram.


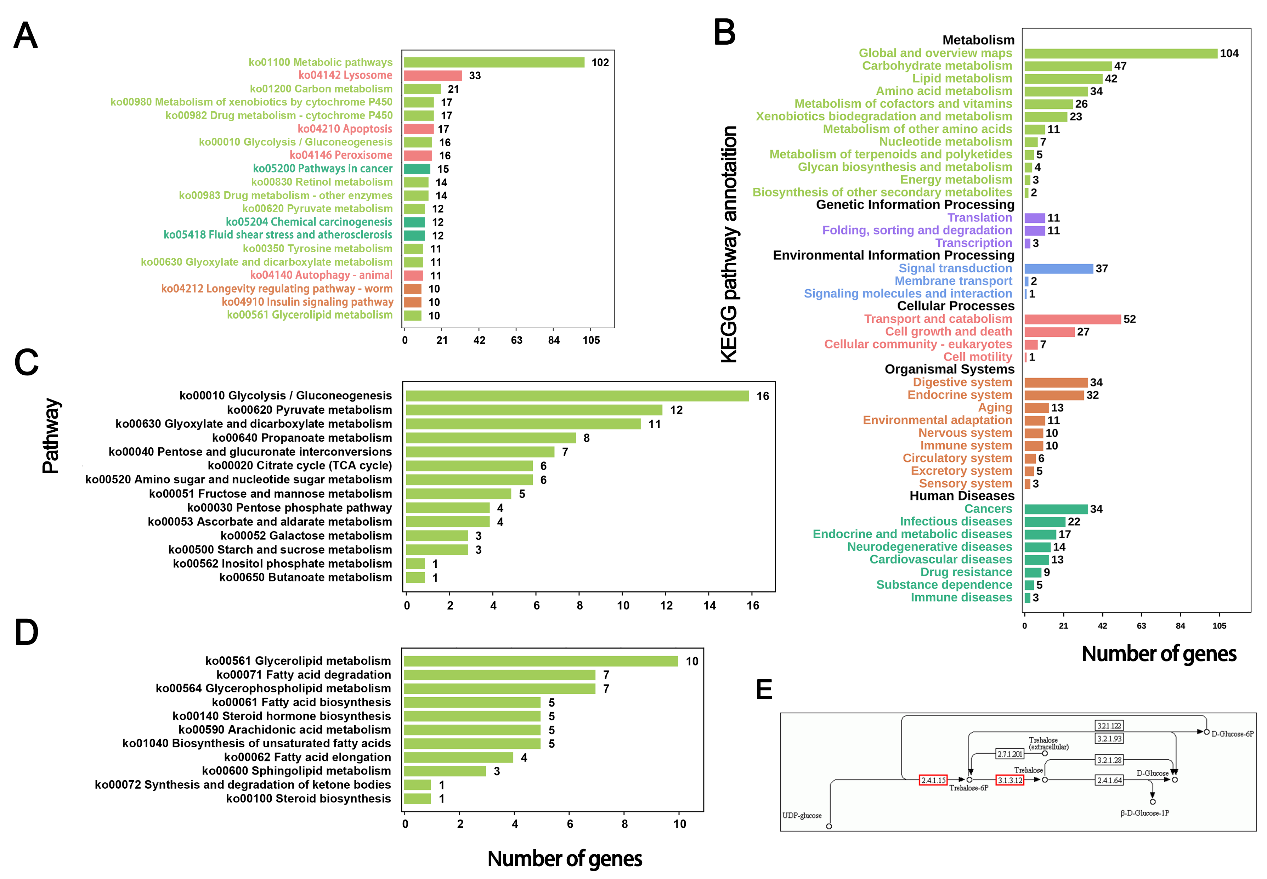


Figure S2. KEGG enrichment analyses of genes in magenta module. **a.** The top 20 pathways with the most enriched genes. **b.** KEGG enrichment analyses. **c.** Enriched pathways in carbohydrate hydration metabolism class. **d.** Enriched pathways in lipid metabolism class. **e.** Reference pathway of trehalose metabolism.


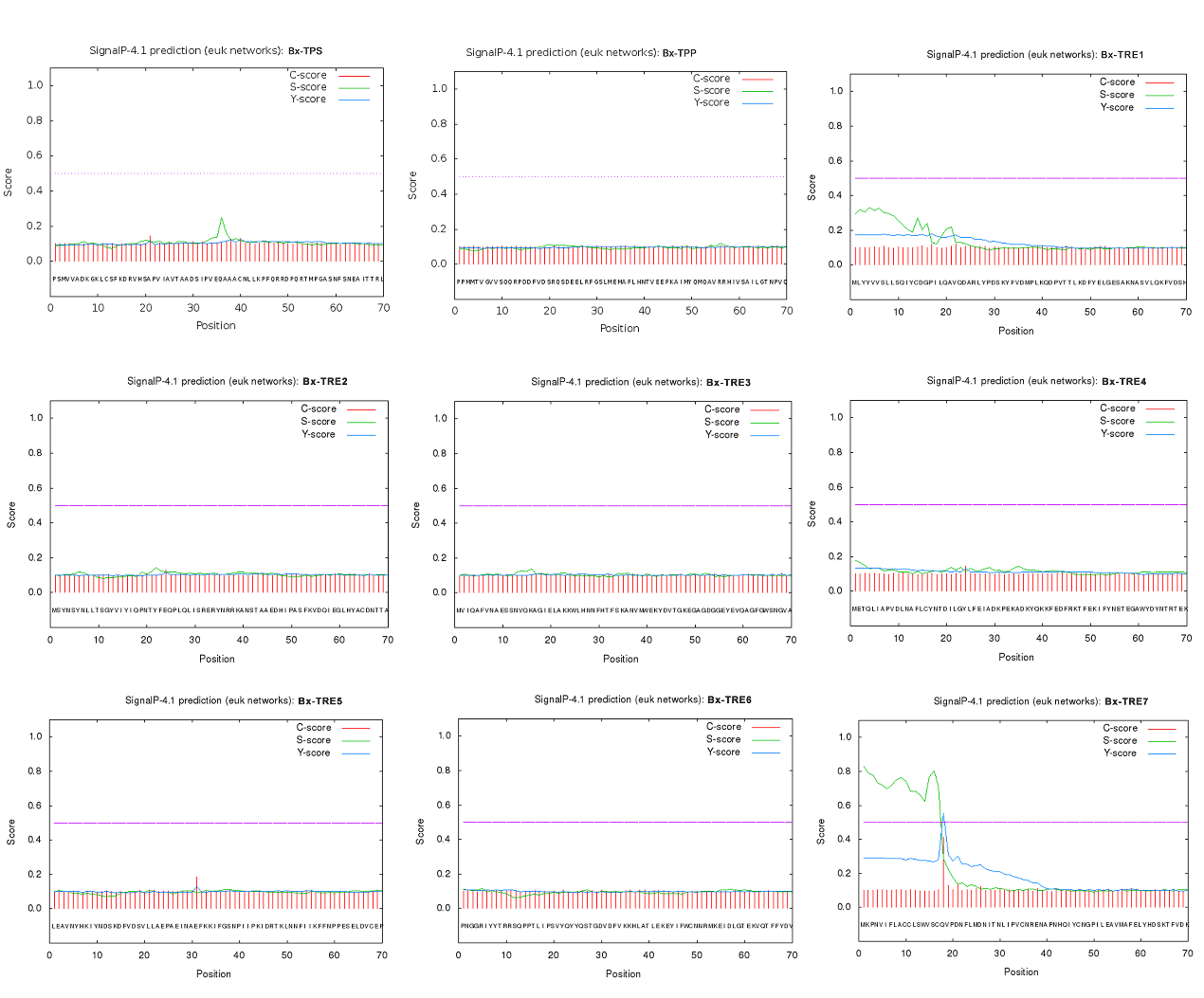


Figure S3. Signal peptide analysis.


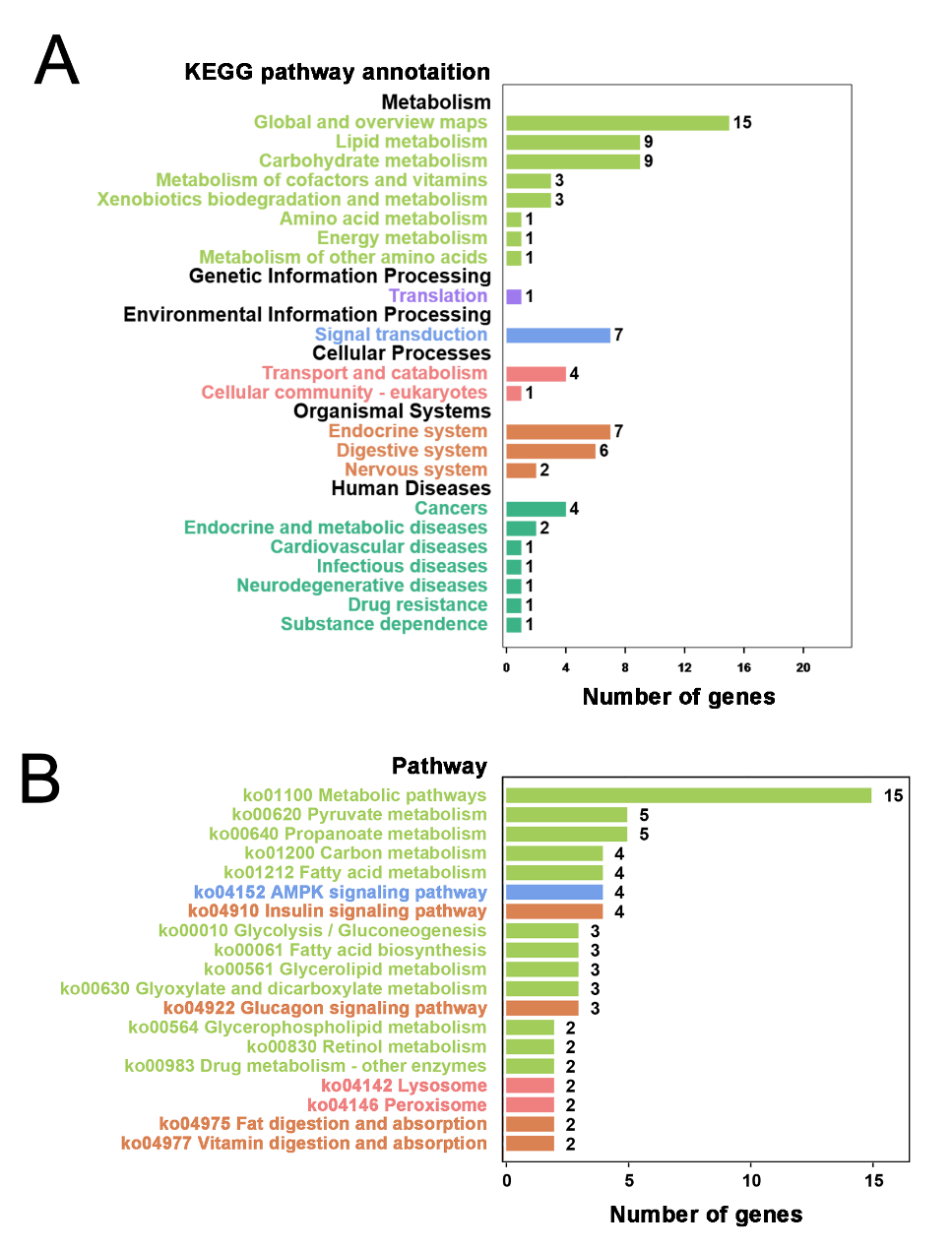


Figure S4. KEGG enrichment analyses of *Bx-tps* and *Bx-tpp* coexpressed genes. **a.** KEGG enrichment analysis for genes highly related to *Bx-tps* and *Bx-tpp* (detailed in Additional file1: Table S9). **b.** The KEGG pathways enriched by most genes.

Table S1. Primers used in cloning encoding sequence.

| Gene | Primer sequence |
| --- | --- |
| *Bx-tps*（BXY_1687600.1） | F：5`-ATGGTGGTGGCAGACAAAG-3` |
|  | R：5`-CTAATCCGACTCTTGGCTGTT-3` |
| *Bx-tpp*（BXY_1399300.1） | F：5`-ATGATGACAGTTGGAGTCGTATC-3` |
|  | R：5`-TTACTTGAGGATTCTCTTTGGCTG-3` |
| *Bx-tre1*（BXY_0921500.1） | F：5`-ATGCTTTACTATGTGGTGTCTTTGT-3` |
|  | R：5`-TCATTCATATATCATCCACATCACTCTG-3` |
| *Bx-tre2*（BXY_0499300.1） | F：5`-ATGTCATATAACTCATATAATCTCCTCACA-3` |
|  | R：5`-CTAAAAAGCGATTCTATTTCTAGGCTT-3` |
| *Bx-tre3*（BXY_0499500.1） | F：5`-ATGGTCATCCAGGCCTTTG-3` |
|  | R：5`-TCAATACAATAGACGTCCTAAGCAG-3` |
| *Bx-tre4*（BXY_0533100.1） | F：5`-ATGGAGACTCAGCTCATCG-3` |
|  | R：5`-TTAAATCAGCTGATAATAAAGATTTATCAATG-3` |
| *Bx-tre5*（BXY_1306200.1） | F：5`-TGGAAGCCGTGAATTACCACA-3` |
|  | R：5`-TTACTCATTAACCATATCGAGCAGA-3` |
| *Bx-tre6*（BXY_1485700.1） | F：5`-CCGAATGGTGGCCGAATTTAC-3` |
|  | R：5`-CGTCATATTGTATTTCTGCGCC-3` |
| *Bx-tre7*（BXY_1733700.1） | F：5`-ATGAAGCCTAATGTAATTTTCTTGGC-3` |
|  | R：5`-CTAAGACGACTCCATTAGTCCAG-3` |

Table S2. Target sequences for siRNA.

| Gene | Target sequence |
| --- | --- |
| *Bx-tps*： | GGA AGA TTA CAA TGA CAA AGC |
| *Bx-tpp*： | GGT CAA TGA TGA GAT GAT AAA |
| *Bx-tre1*： | GAG TGA TGT GGA TGA TAT ATG |
| *Bx-tre2*： | GGC GAT TCC GAG AGT TCT ACT |
| *Bx-tre3*： | GCT TAG GAC GTC TAT TGT ATT |
| *Bx-tre4*： | GGA GTT GTA TTG GAC ATA ATG |
| *Bx-tre5*： | GGA GAT TCC GAG AGT TCT ATT |
| *Bx-tre6*： | GGT GCA ACA ACC GAA TGA AGG |
| *Bx-tre7*： | GGA TAA CAT CAC TAA TCT AAT |
| nt siRNA： | AGG AGC TGT TCA CCG GGG TGG |

Table S3. Primers used in RT-qPCR.

| Gene | Primer sequence |
| --- | --- |
| *Bx-tps*（BXY_1687600.1） | F：5`-GGACCCGAACACGGAAGATT-3` |
|  | R：5`-GATAGCCGAGGGCATTGACA-3` |
| *Bx-tpp*（BXY_1399300.1） | F：5`-GGGAAATGCTGGCACCTAGT-3` |
|  | R：5`-CAGAGACGGGGTGATATGGC-3` |
| *Bx-tre1*（BXY_0921500.1） | F：5`-TTCACAAGACCGCCCGATAC-3` |
|  | R：5`-GAGAACACCTTCCCGCTTCA-3` |
| *Bx-tre2*（BXY_0499300.1） | F：5`-AACATCCGTTCGTCGTTCCA-3` |
|  | R：5`-AGCGAGAAGTGGAGGTTGTG-3` |
| *Bx-tre3*（BXY_0499500.1） | F：5`-CGATGTGACGGGCAAAGAAG-3` |
|  | R：5`-TCGCTATGGAAGACAAACGCT-3` |
| *Bx-tre4*（BXY_0533100.1） | F：5`-ATTGCGGACAAACCGGAGAA-3` |
|  | R：5`-TTTGCTCTCGTCCAAGCGAT-3` |
| *Bx-tre5*（BXY_1306200.1） | F：5`-TGGAAGCCGTGAATTACCACA-3` |
|  | R：5`-GGCTCACAAACGTCCAACTC-3` |
| *Bx-tre6*（BXY_1485700.1） | F：5`-CCGAATGGTGGCCGAATTTAC-3` |
|  | R：5`-CTTGGGACGTTCGTTGGGA-3` |
| *Bx-tre7*（BXY_1733700.1） | F：5`-ATCGTCCGAAACGAAAGCGA-3` |
|  | R：5`-AATAGTGGCGGCTGTGATCG-3` |
| *28s RNA* | F：5`-CAGCTTTGTGGAGACGTGG-3` |
|  | R：5`-GAAGAACGCAGAGCACACC-3` |

Table S4. Statistical analysis of the RNA sequencing data.

| Samples | Clean Reads Number | Clean Reads Rate (%) | Clean Reads Q20 (%) | Gene Mapping Rate (%) |
| --- | --- | --- | --- | --- |
| J2-1 | 22,133,728 | 99.62 | 97.50 | 71.27 |
| J2-2 | 22,203,673 | 99.94 | 97.38 | 71.64 |
| J2-3 | 24,112,070 | 99.90 | 97.94 | 71.58 |
| J3-1 | 24,057,741 | 99.67 | 97.20 | 75.77 |
| J3-2 | 24,145,341 | 99.56 | 97.64 | 74.66 |
| J3-3 | 23,985,362 | 99.48 | 97.67 | 75.13 |
| J4-1 | 24,037,515 | 99.59 | 96.90 | 76.96 |
| J4-2 | 23,465,363 | 99.65 | 97.64 | 76.68 |
| J4-3 | 24,013,521 | 99.56 | 97.68 | 76.84 |
| J2-2-1 | 24,110,169 | 99.89 | 97.90 | 59.92 |
| J2-2-2 | 23,951,367 | 99.39 | 97.86 | 64.57 |
| J2-2-3 | 24,061,351 | 99.86 | 97.34 | 62.44 |
| DJ3-1 | 24,018,211 | 99.51 | 97.10 | 75.94 |
| DJ3-2 | 23,996,384 | 99.42 | 97.00 | 74.01 |
| DJ3-3 | 23,940,211 | 99.80 | 98.40 | 73.51 |
| female-1 | 23,960,379 | 99.26 | 95.30 | 78.06 |
| female-2 | 24,013,135 | 99.46 | 97.34 | 78.99 |
| female-3 | 23,961,244 | 99.75 | 97.42 | 78.56 |
| male-1 | 21,359,122 | 99.41 | 94.80 | 76.86 |
| male-2 | 22,063,153 | 99.53 | 96.57 | 76.96 |
| male-3 | 21,624,233 | 99.75 | 97.31 | 76.66 |

Table S5. Alignment results of trehalose metabolism related genes in pine wood nematode.

| *C. elegans* gene | *C. elegans* gene ID | PWN gene ID | identity（%） | alignment length | start | end | E-value | bit score |
| --- | --- | --- | --- | --- | --- | --- | --- | --- |
| *tps1* | AJ512332.1 | BXY_1687600.1 | 55 | 1000 | 958 | 3873 | 0 | 1153 |
|  | AJ512332.1 | BXY_1687600.1 | 52.066 | 121 | 448 | 810 | 4.22E-35 | 145 |
|  | AJ512332.1 | BXY_1399300.1 | 25.77 | 357 | 2644 | 3657 | 7.01E-27 | 114 |
| *tps2* | AJ512333.1 | BXY_1687600.1 | 61.519 | 1211 | 207 | 3665 | 0 | 1518 |
|  | AJ512333.1 | BXY_1399300.1 | 27.324 | 355 | 2433 | 3431 | 7.05E-36 | 140 |
| *tpp* | NM_078159.5 | BXY_1399300.1 | 46.629 | 356 | 91 | 1143 | 3.66E-108 | 331 |
|  | NM_078159.5 | BXY_1687600.1 | 29.255 | 376 | 307 | 1422 | 4.35E-40 | 157 |
| *tre1* | AJ512337.1 | BXY_0921500.1 | 66.004 | 503 | 1 | 1503 | 0 | 706 |
|  | AJ512337.1 | BXY_1733700.1 | 40.984 | 488 | 31 | 1485 | 2.25E-132 | 399 |
|  | AJ512337.1 | BXY_0499300.1 | 40.509 | 432 | 34 | 1311 | 3.86E-111 | 343 |
|  | AJ512337.1 | BXY_1306200.1 | 45.355 | 183 | 25 | 546 | 1.76E-45 | 159 |
|  | AJ512337.1 | BXY_0533100.1 | 39.819 | 221 | 958 | 1617 | 7.26E-39 | 143 |
|  | AJ512337.1 | BXY_1485700.1 | 54.255 | 94 | 553 | 831 | 4.27E-25 | 100 |
| *tre2* | AJ512334.1 | BXY_0921500.1 | 42.137 | 496 | 245 | 1699 | 3.17E-133 | 402 |
|  | AJ512334.1 | BXY_1733700.1 | 40.741 | 486 | 251 | 1672 | 7.84E-120 | 368 |
|  | AJ512334.1 | BXY_0499300.1 | 39.352 | 432 | 251 | 1516 | 3.28E-97 | 308 |
|  | AJ512334.1 | BXY_0533100.1 | 32.78 | 241 | 1175 | 1870 | 1.79E-38 | 142 |
|  | AJ512334.1 | BXY_1306200.1 | 38.889 | 180 | 236 | 763 | 2.54E-36 | 134 |
|  | AJ512334.1 | BXY_1485700.1 | 41.667 | 96 | 770 | 1048 | 4.31E-15 | 71.6 |
| *tre3* | AJ512335.1 | BXY_1733700.1 | 52.016 | 496 | 131 | 1558 | 0 | 551 |
|  | AJ512335.1 | BXY_0921500.1 | 43.173 | 498 | 164 | 1576 | 9.80E-143 | 424 |
|  | AJ512335.1 | BXY_0499300.1 | 43.146 | 445 | 134 | 1393 | 6.10E-125 | 378 |
|  | AJ512335.1 | BXY_0533100.1 | 53.604 | 222 | 1028 | 1693 | 6.81E-87 | 270 |
|  | AJ512335.1 | BXY_1306200.1 | 40.212 | 189 | 134 | 637 | 1.45E-41 | 148 |
|  | AJ512335.1 | BXY_1485700.1 | 41.284 | 109 | 590 | 916 | 9.88E-23 | 93.6 |
|  | AJ512335.1 | BXY_0499500.1 | 43.373 | 83 | 1442 | 1690 | 1.28E-17 | 78.6 |
| *tre4* | AJ512338.1 | BXY_1733700.1 | 34.331 | 501 | 130 | 1554 | 1.06E-87 | 282 |
|  | AJ512338.1 | BXY_0921500.1 | 34.861 | 502 | 157 | 1590 | 5.14E-85 | 275 |
|  | AJ512338.1 | BXY_0499300.1 | 35.047 | 428 | 166 | 1377 | 2.13E-75 | 249 |
|  | AJ512338.1 | BXY_0533100.1 | 32.11 | 218 | 1033 | 1674 | 2.03E-33 | 127 |
|  | AJ512338.1 | BXY_1306200.1 | 33.708 | 178 | 157 | 660 | 9.10E-21 | 90.1 |
|  | AJ512338.1 | BXY_1485700.1 | 36.082 | 97 | 652 | 924 | 6.79E-15 | 70.9 |
| *tre5* | AJ512339.1 | BXY_1733700.1 | 50.501 | 499 | 233 | 1729 | 0 | 535 |
|  | AJ512339.1 | BXY_0499300.1 | 46.868 | 463 | 206 | 1585 | 3.08E-150 | 445 |
|  | AJ512339.1 | BXY_0921500.1 | 42.315 | 501 | 266 | 1747 | 9.31E-144 | 429 |
|  | AJ512339.1 | BXY_0533100.1 | 49.321 | 221 | 1199 | 1861 | 3.11E-75 | 241 |
|  | AJ512339.1 | BXY_1306200.1 | 39.326 | 178 | 266 | 790 | 1.41E-35 | 132 |
|  | AJ512339.1 | BXY_1485700.1 | 44.444 | 99 | 797 | 1090 | 1.00E-20 | 87.8 |
|  | AJ512339.1 | BXY_0499500.1 | 46.341 | 82 | 1613 | 1858 | 1.95E-18 | 80.9 |

Table S6. Expression of trehalose metabolism related genes after RNAi treatment.

| RNAi  treatment | Log_2_(RNAi/CK2)^*^ | | | | | | | | |
| --- | --- | --- | --- | --- | --- | --- | --- | --- | --- |
|  | *Bx-tps* | *Bx-tpp* | *Bx-tre1* | *Bx-tre2* | *Bx-tre3* | *Bx-tre4* | *Bx-tre5* | *Bx-tre6* | *Bx-tre7* |
| *Bx-tps* | −1.87  ±0.07 | 0.27  ±0.12 | -0.12  ±0.03 | 0.25  ±0.24 | -0.57  ±0.05 | 0.36  ±0.34 | -0.53  ±0.04 | 0.12  ±0.12 | -0.15  ±0.24 |
| *Bx-tpp* | -0.24  ±0.04 | −1.48  ±0.30 | 0.52  ±0.15 | -0.42  ±0.32 | 0.19  ±0.06 | -0.45  ±0.33 | -0.23  ±0.11 | 0.37  ±0.12 | -0.42  ±0.32 |
| *Bx-tre1* | 0.24  ±0.25 | -0.37  ±0.32 | −1.34  ±0.39 | -0.42  ±0.15 | 0.23  ±0.29 | 0.19  ±0.22 | -0.02  ±0.13 | 0.07  ±0.23 | -0.42  ±0.15 |
| *Bx-tre2* | -0.23  ±0.22 | 0.54  ±0.19 | -0.22  ±0.32 | −1.75  ±0.10 | 0.42  ±0.11 | -0.44  ±0.12 | 0.33  ±0.23 | 0.19  ±0.12 | -0.08  ±0.24 |
| *Bx-tre3* | 0.13  ±0.10 | -0.25  ±0.12 | -0.42  ±0.32 | 0.37  ±0.11 | −1.39  ±0.07 | -0.19  ±0.09 | 0.34  ±0.13 | -0.42  ±0.12 | 0.21  ±0.13 |
| *Bx-tre4* | -0.31  ±0.13 | 0.24  ±0.23 | 0.28  ±0.31 | -0.37  ±0.24 | 0.19  ±0.16 | −1.44  ±0.05 | -0.06  ±0.14 | 0.04  ±0.23 | -0.16  ±0.03 |
| *Bx-tre5* | 0.15  ±0.11 | -0.42  ±0.01 | 0.19  ±0.02 | -0.08  ±0.04 | 0.01  ±0.22 | 0.21  ±0.11 | −1.57  ±0.05 | 0.43  ±0.19 | 0.18  ±0.09 |
| *Bx-tre6* | 0.22  ±0.14 | 0.31  ±0.22 | -0.33  ±0.11 | 0.25  ±0.12 | -0.19  ±0.07 | 0.07  ±0.18 | -0.02  ±0.32 | −1.39  ±0.06 | 0.45  ±0.11 |
| *Bx-tre7* | 0.42  ±0.17 | -0.05  ±0.23 | 0.07  ±0.26 | -0.15  ±0.31 | 0.28  ±0.08 | 0.33  ±0.06 | -0.29  ±0.12 | 0.19  ±0.13 | −1.49  ±0.07 |

^*^Data are given as means±s.d.; N=3.

Table S7. Expression of trehalose metabolism related genes after RNAi treatment.

| RNAi  treatment | Log_2_(RNAi/CK3)^*^ | | | | | | | | |
| --- | --- | --- | --- | --- | --- | --- | --- | --- | --- |
|  | *Bx-tps* | *Bx-tpp* | *Bx-tre1* | *Bx-tre2* | *Bx-tre3* | *Bx-tre4* | *Bx-tre5* | *Bx-tre6* | *Bx-tre7* |
| *Bx-tps* | −1.77  ±0.17 | 0.23  ±0.11 | -0.33  ±0.04 | 0.14  ±0.14 | -0.46  ±0.04 | 0.36  ±0.34 | -0.43  ±0.04 | 0.11  ±0.13 | -0.14  ±0.14 |
| *Bx-tpp* | -0.14  ±0.24 | −1.46  ±0.13 | 0.33  ±0.05 | -0.41  ±0.31 | 0.19  ±0.06 | -0.44  ±0.33 | -0.13  ±0.11 | 0.36  ±0.11 | -0.41  ±0.31 |
| *Bx-tre1* | 0.42  ±0.11 | -0.43  ±0.11 | −1.54  ±0.23 | -0.41  ±0.14 | 0.13  ±0.19 | 0.19  ±0.11 | -0.01  ±0.13 | 0.06  ±0.13 | -0.41  ±0.14 |
| *Bx-tre2* | -0.34  ±0.13 | 0.34  ±0.03 | -0.13  ±0.35 | −1.64  ±0.10 | 0.41  ±0.11 | -0.44  ±0.11 | 0.33  ±0.13 | 0.19  ±0.11 | -0.08  ±0.14 |
| *Bx-tre3* | 0.23  ±0.04 | -0.38  ±0.13 | -0.13  ±0.19 | 0.36  ±0.11 | −1.79  ±0.06 | -0.19  ±0.09 | 0.34  ±0.13 | -0.41  ±0.11 | 0.11  ±0.13 |
| *Bx-tre4* | -0.23  ±0.14 | 0.11  ±0.13 | 0.35  ±0.13 | -0.36  ±0.14 | 0.19  ±0.16 | −1.66  ±0.04 | -0.06  ±0.14 | 0.04  ±0.13 | -0.16  ±0.03 |
| *Bx-tre5* | 0.36  ±0.02 | -0.22  ±0.10 | 0.43  ±0.08 | -0.08  ±0.04 | 0.01  ±0.11 | 0.11  ±0.11 | −1.58  ±0.04 | 0.43  ±0.19 | 0.18  ±0.09 |
| *Bx-tre6* | 0.34  ±0.09 | 0.13  ±0.02 | -0.46  ±0.03 | 0.14  ±0.11 | -0.19  ±0.06 | 0.06  ±0.18 | -0.01  ±0.31 | −1.47  ±0.06 | 0.44  ±0.11 |
| *Bx-tre7* | 0.43  ±0.18 | -0.43  ±0.33 | 0.11  ±0.16 | -0.14  ±0.31 | 0.18  ±0.08 | 0.33  ±0.06 | -0.19  ±0.11 | 0.19  ±0.13 | −1.78  ±0.06 |

^*^Data are given as means±s.d.; N=3.

Table S8. Selected genes in the magenta module with high weight value (>0.2) to *Bx-tps* or *Bx-tpp*.

| No. | log_2_fold | GS | MM | IC |
| --- | --- | --- | --- | --- |
| 1 | 4.13093087 | 0.995251645 | 0.981177627 | 190.4327015 |
| 2 | 5.863474153 | 0.998394217 | 0.977489615 | 188.5344519 |
| 3 | 4.692186966 | 0.986719719 | 0.981869596 | 188.042847 |
| 4 | 4.163278813 | 0.980256789 | 0.981038279 | 187.6929047 |
| 5 | 3.897619407 | 0.995687417 | 0.978649511 | 187.6237443 |
| 6 | 3.134465113 | 0.992619472 | 0.973740556 | 183.7805316 |
| 7 | 5.694806168 | 0.995042955 | 0.972989156 | 181.8556233 |
| 8 | 1.609797984 | 0.943794643 | 0.975874532 | 174.9462569 |
| 9 | 2.045863371 | 0.96382183 | 0.977044492 | 173.9518743 |
| 10 | 1.883526603 | 0.975191278 | 0.961462892 | 170.4655206 |
| 11 | 2.20382044 | 0.951593236 | 0.971298773 | 170.1207765 |
| 12 | 2.843408101 | 0.97859612 | 0.963699583 | 168.6300303 |
| 13 | 2.436511085 | 0.959836934 | 0.964657005 | 167.191898 |
| 14 | 4.222299497 | 0.979965408 | 0.953742928 | 162.2854186 |
| 15 | 2.511423052 | 0.963959411 | 0.956823191 | 159.9876783 |
| 16 | 2.683532544 | 0.964456332 | 0.957453527 | 159.8079098 |
| 17 | 3.594929345 | 0.966401194 | 0.95715068 | 158.876366 |
| 18 | 2.060644141 | 0.964023804 | 0.952864799 | 158.8046461 |
| 19 | 2.515662864 | 0.943875801 | 0.95278213 | 158.4373088 |
| 20 | 1.73881639 | 0.965273498 | 0.954028649 | 157.510319 |
| 21 | 3 | 0.940790846 | 0.962474071 | 156.7115091 |
| 22 | 3.333113718 | 0.957270894 | 0.95160879 | 156.529679 |
| 23 | 1.492740957 | 0.942097063 | 0.955229747 | 152.9923961 |
| 24 | 3.421336316 | 0.93136121 | 0.947842905 | 152.781391 |
| 25 | 2.053260757 | 0.953027881 | 0.94726407 | 152.5966472 |
| 26 | 2.081650289 | 0.979567873 | 0.942055097 | 151.6243368 |
| 27 | 2.769637338 | 0.963577964 | 0.947139886 | 150.7688246 |
| 28 | 1.948605017 | 0.923548755 | 0.942252639 | 148.4957157 |
| 29 | 1.6180483 | 0.93019346 | 0.952960457 | 148.4685639 |
| 30 | 2.434379229 | 0.915050414 | 0.950546783 | 148.2092388 |
| 31 | 2.422292444 | 0.954272948 | 0.945396566 | 146.704121 |
| 32 | 2.814057999 | 0.96547795 | 0.936250844 | 145.2168671 |
| 33 | 1.570005659 | 0.926600556 | 0.937599078 | 145.0719155 |
| 34 | 1.222583857 | 0.91465526 | 0.943230558 | 140.6763026 |
| 35 | 1.43702448 | 0.943398082 | 0.942382539 | 138.8591108 |
| 36 | 2.265607545 | 0.945734637 | 0.930663599 | 136.6759908 |
| 37 | 1.81651997 | 0.915761026 | 0.931107198 | 136.5442672 |
| 38 | 1.475223452 | 0.938272304 | 0.933588541 | 134.4763626 |
| 39 | 1.367590337 | 0.957343292 | 0.922915508 | 134.2656089 |
| 40 | 1.357179421 | 0.913457344 | 0.925431122 | 133.676126 |
| 41 | 1.454647424 | 0.89620595 | 0.945075812 | 133.5671049 |
| 42 | 2.207752586 | 0.899506352 | 0.929412936 | 132.2186433 |
| 43 | 2.547920799 | 0.952702267 | 0.925440672 | 129.8689203 |
| 44 | 1.819258712 | 0.891410108 | 0.927737805 | 129.5577643 |
| 45 | 2.162601938 | 0.916452648 | 0.932391447 | 128.7327547 |
| 46 | 4.190169507 | 0.948994249 | 0.918076114 | 128.4053232 |
| 47 | 2.832251795 | 0.915748687 | 0.924876876 | 127.8668765 |
| 48 | 2.413774869 | 0.95128171 | 0.91816933 | 127.0329382 |
| 49 | 2.015222319 | 0.920877526 | 0.921957625 | 125.6100861 |
| 50 | 1.72102405 | 0.947965201 | 0.910518249 | 125.5190891 |
| 51 | 0.934995361 | 0.914299255 | 0.914721406 | 123.4643279 |
| 52 | 2.542855107 | 0.886211437 | 0.917917309 | 123.3213065 |
| 53 | 1.961735092 | 0.862433742 | 0.912322155 | 121.577259 |
| 54 | 2.403789531 | 0.88002777 | 0.914321392 | 121.149594 |
| 55 | 2.241670013 | 0.908577312 | 0.903287898 | 120.239645 |
| 56 | 1.852907908 | 0.859583616 | 0.921862218 | 118.4909139 |
| 57 | 2.320982209 | 0.901458891 | 0.902211154 | 114.9459835 |
| 58 | 1.885804836 | 0.847275852 | 0.909273107 | 114.378455 |
| 59 | 1.378260468 | 0.846896034 | 0.917822758 | 113.9905916 |
| 60 | 2.056666291 | 0.895678407 | 0.901827405 | 113.2217923 |
| 61 | 3.286029069 | 0.837468594 | 0.91387454 | 110.6530932 |
| 62 | 1.494965669 | 0.883868514 | 0.896537658 | 109.5469357 |
| 63 | 2.313576014 | 0.891707182 | 0.899560402 | 109.1620679 |
| 64 | 1.216500255 | 0.910494403 | 0.894482989 | 108.8865173 |
| 65 | 1.684583061 | 0.913639643 | 0.887104056 | 108.5789342 |
| 66 | 2.128297906 | 0.893435012 | 0.909809874 | 108.2957805 |
| 67 | 1.783351696 | 0.903416512 | 0.894430849 | 107.7896535 |
| 68 | 1.097555522 | 0.882025485 | 0.892677235 | 106.7228991 |
| 69 | 2.396434909 | 0.889620728 | 0.890315949 | 106.2821821 |
| 70 | 1.381979008 | 0.862625417 | 0.911208711 | 105.2562552 |
| 71 | 1.508629488 | 0.869054251 | 0.899043369 | 105.0785553 |
| 72 | 2.45042467 | 0.923630305 | 0.900248096 | 104.4207377 |
| 73 | 1.148555054 | 0.884342446 | 0.889521363 | 103.6840211 |
| 74 | 1.407462187 | 0.862028266 | 0.895883541 | 102.509495 |
| 75 | 2.664198138 | 0.893198462 | 0.892625898 | 101.2641833 |
| 76 | 1.154700672 | 0.846074237 | 0.91040337 | 100.2186998 |
| 77 | 1.033178877 | 0.901698277 | 0.884292974 | 99.06631022 |
| 78 | 2.00062531 | 0.922579001 | 0.870702007 | 95.93057172 |
| 79 | 1.412312862 | 0.798794083 | 0.888931342 | 94.00137299 |
| 80 | 1.392546659 | 0.822965632 | 0.89529022 | 92.54298455 |
| 81 | 2.289181313 | 0.864354809 | 0.869707735 | 91.13947672 |
| 82 | 0.965754615 | 0.855013788 | 0.882562456 | 89.95871787 |
| 83 | 2.275593348 | 0.789111233 | 0.887640822 | 88.64836511 |
| 84 | 1.23937721 | 0.800578323 | 0.889555744 | 85.39207329 |
| 85 | 2.624780324 | 0.828340634 | 0.855476629 | 84.11860118 |
| 86 | 1.234690084 | 0.861298738 | 0.860862487 | 83.67617952 |
| 87 | 3.895429597 | 0.890042872 | 0.839990548 | 77.82561428 |
| 88 | 1.741265732 | 0.862434056 | 0.8353446 | 74.28792164 |
| 89 | 1.308815264 | 0.835896996 | 0.854934679 | 71.04967022 |
| 90 | 0.75468386 | 0.871403092 | 0.834398979 | 69.33933993 |
| 91 | 1.221011706 | 0.766181931 | 0.851377613 | 67.16742789 |
| 92 | 1.362950603 | 0.811111223 | 0.846366098 | 65.38059524 |
| 93 | -1.07153870 | 0.835374478 | -0.83888073 | 59.17958502 |
| 94 | 1.262632907 | 0.865511848 | 0.804615628 | 57.6098505 |
| 95 | 1.393033346 | 0.704151465 | 0.804464483 | 56.72460679 |
| 96 | 1.794178181 | 0.802082178 | 0.789809762 | 54.06276479 |
| 97 | 0.661410215 | 0.736961773 | 0.780655952 | 48.37894997 |
| 98 | 0.656510033 | 0.741224966 | 0.779215375 | 39.02729722 |
| 99 | 1.185851798 | 0.675702388 | 0.743636422 | 32.86456421 |

Table S9. KEGG enrichment for genes highly related to *Bx-tps1* and *Bx-tps2*.

| KEGG A class | KEGG B class | Pathway | Amount | Pathway ID | Gene No. | K IDs |
| --- | --- | --- | --- | --- | --- | --- |
| Metabolism | Carbohydrate metabolism | Pyruvate metabolism | 5 | ko00620 | 42，30，13，6，32 | K11262，K11262，K11262，K01895，K01895 |
| Metabolism | Carbohydrate metabolism | Propanoate metabolism | 5 | ko00640 | 42，30，13，6，32 | K11262，K11262，K11262，K01895，K01895 |
| Metabolism | Global and overview maps | Carbon metabolism | 4 | ko01200 | 6，32，62，8 | K01895，K01895，K01647，K00036 |
| Metabolism | Global and overview maps | Fatty acid metabolism | 4 | ko01212 | 42，30，13，96 | K11262，K11262，K11262，K10203 |
| Environmental Information Processing | Signal transduction | AMPK signaling pathway | 4 | ko04152 | 42，30，13，55 | K11262，K11262，K11262，K07197 |
| Organismal Systems | Endocrine system | Insulin signaling pathway | 4 | ko04910 | 42，30，13，55 | K11262，K11262，K11262，K07197 |
| Metabolism | Carbohydrate metabolism | Glycolysis / Gluconeogenesis | 3 | ko00010 | 27，6，32 | K13953，K01895，K01895 |
| Metabolism | Lipid metabolism | Fatty acid biosynthesis | 3 | ko00061 | 42，30，13 | K11262，K11262，K11262 |
| Metabolism | Lipid metabolism | Glycerolipid metabolism | 3 | ko00561 | 98，12，86 | K14458，K14457，K13509 |
| Metabolism | Carbohydrate metabolism | Glyoxylate and dicarboxylate metabolism | 3 | ko00630 | 6，32，62 | K01895，K01895，K01647 |
| Organismal Systems | Endocrine system | Glucagon signaling pathway | 3 | ko04922 | 42，30，13 | K11262，K11262，K11262 |
| Metabolism | Lipid metabolism | Glycerophospholipid metabolism | 2 | ko00564 | 86，89 | K13509，K00111 |
| Cellular Processes | Transport and catabolism | Lysosome | 2 | ko04142 | 66，51 | K12385，K12307 |
| Cellular Processes | Transport and catabolism | Peroxisome | 2 | ko04146 | 97，72 | K13336，K11147 |
| Organismal Systems | Digestive system | Fat digestion and absorption | 2 | ko04975 | 12，86 | K14457，K13509 |

Table S10. Gene annotation result.

| Gene No. | nr ID | E-value | Annotation |
| --- | --- | --- | --- |
| 6 | KJH44216.1 | 1.21E-75 | acetate--CoA ligase |
| 8 | KHN74130.1 | 0 | Glucose-6-phosphate 1-dehydrogenase |
| 12 | CAP39053.1 | 1.79E-59 | Protein CBG22471 |
| 13 | CEF71468.1 | 0 | Carboxyl transferase domain and Biotin/lipoyl attachment domain and Carbamoyl-phosphate synthetase large subunit-like, ATP-binding domain and Carbamoyl-phosphate synthase, large subunit, N-terminal domain and Biotin carboxylase, C-terminal |
| 27 | XP_002636550.1 | 5.40E-102 | *C. briggsae* CBR-SODH-1 protein |
| 30 | KKA67036.1 | 4.27E-14 | pod-2 |
| 32 | EJW73448.1 | 3.02E-23 | acetyl-CoA synthetase |
| 42 | CEF71468.1 | 1.08E-169 | Carboxyl transferase domain and Biotin/lipoyl attachment domain and Carbamoyl-phosphate synthetase large subunit-like, ATP-binding domain and Carbamoyl-phosphate synthase, large subunit, N-terminal domain and Biotin carboxylase, C-terminal |
| 51 | CEF66825.1 | 9.98E-101 | Major facilitator superfamily and Major facilitator superfamily domain, general substrate transporter and Major facilitator superfamily domain-containing protein |
| 55 | KHN86442.1 | 3.25E-87 | Sterol regulatory element-binding protein 1 |
| 62 | AFL48196.1 | 0 | citrate synthase, partial |
| 66 | KHN70676.1 | 7.50E-114 | Patched domain-containing protein 3 |
| 72 | CEF69669.1 | 9.41E-44 | Short-chain dehydrogenase/reductase SDR family and Glucose/ribitol dehydrogenase family and NAD(P)-binding domain-containing protein |
| 86 | XP_013304900.1 | 1.43E-54 | Acyltransferase |
| 89 | EPB72861.1 | 0 | FAD dependent oxidoreductase |
| 96 | CDJ84522.1 | 2.16E-88 | GNS1 SUR4 membrane protein domain containing protein |
| 97 | ERG81975.1 | 3.16E-22 | peroxisomal biogenesis factor 3 |
| 98 | ERG81224.1 | 9.91E-68 | 2-acylglycerol o-acyltransferase 2-a |
